# Real-Time Visual Anomaly Detection in High-Speed Motorsport: An Entropy-Driven Hybrid Retrieval- and Cache-Augmented Architecture

**DOI:** 10.3390/jimaging12020060

**Published:** 2026-01-28

**Authors:** Rubén Juárez Cádiz, Fernando Rodríguez-Sela

**Affiliations:** 1 Engineering School, CEU San Pablo University, Campus de Montepríncipe, Av. de Montepríncipe, s/n, 28925 Madrid, Spain; 2 School of Engineering, Science, and Technology, UNIE Universidad, Calle Arapiles, 28015 Madrid, Spain

**Keywords:** real-time computer vision, visual anomaly detection, tail-latency-aware inference, cache-augmented inference, similarity retrieval, telemetry–vision fusion, edge AI, uncertainty estimation, entropy-based routing, motorsport imaging

## Abstract

At 300 km/h, an end-to-end vision delay of 100 ms corresponds to 8.3 m of unobserved travel; therefore, real-time anomaly monitoring must balance sensitivity with strict tail-latency constraints at the edge. We propose a hybrid cache–retrieval inference architecture for visual anomaly detection in high-speed motorsport that exploits lap-to-lap spatiotemporal redundancy while reserving local similarity retrieval for genuinely uncertain events. The system combines a hierarchical visual encoder (a lightweight backbone with selective refinement via a Nested U-Net for texture-level cues) and an uncertainty-driven router that selects between two memory pathways: (i) a static cache of precomputed scene embeddings for track/background context and (ii) local similarity retrieval over historical telemetry–vision patterns to ground ambiguous frames, improve interpretability, and stabilize decisions under high uncertainty. Routing is governed by an entropy signal computed from prediction and embedding uncertainty: low-entropy frames follow a cache-first path, whereas high-entropy frames trigger retrieval and refinement to preserve decision stability without sacrificing latency. On a high-fidelity closed-circuit benchmark with synchronized onboard video and telemetry and controlled anomaly injections (tire degradation, suspension chatter, and illumination shifts), the proposed approach reduces mean end-to-end latency to 21.7 ms versus 48.6 ms for a retrieval-only baseline (55.3% reduction) while achieving Macro-F1 = 0.89 at safety-oriented operating points. The framework is designed for passive monitoring and decision support, producing advisory outputs without actuating ECU control strategies.

## 1. Introduction

Premier-class motorcycle racing is approaching a major technical transition with the MotoGP 2027 package, which reduces displacement to 850 cc (with a 75 mm maximum bore), restricts aerodynamic appendages, and prohibits mechanical ride-height and holeshot devices [1,2,3]. Beyond performance, these constraints reshape load transfer and chassis attitude management, increasing the operational relevance of early indicators of oscillatory instabilities (e.g., headshake, braking-induced vibration modes, and chassis–suspension coupling). Such phenomena have been studied extensively in the racing context under the umbrella of chatter and related self-excited vibration modes [4,5,6,7].

**Figure 1:**
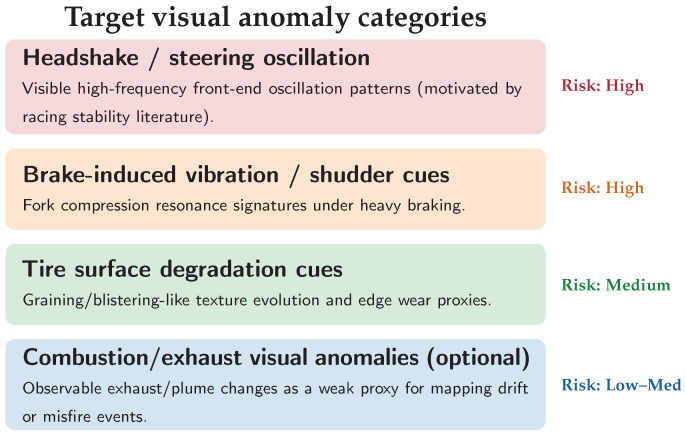
Target anomaly classes. Representative visual anomaly categories addressed in this paper.

These dynamics motivate *visual* monitoring as a complementary modality. Standard telemetry channels (IMU, suspension travel, wheel speeds) remain indispensable for quantitative state estimation, but high-resolution onboard video can expose cues that are difficult to capture directly (e.g., tire surface texture evolution, subtle oscillation patterns in the front assembly, or transient signatures under illumination shifts). Prior work in multimodal learning and motorsport data analysis supports the view that fusing heterogeneous signals can improve disambiguation between aggressive maneuvers and failure precursors [8,9]. Likewise, vision-based anomaly detection indicates that convolutional features can capture fine-grained texture patterns that may precede macroscopic failures [10,11]. In this paper, we focus on safety-relevant visual categories such as steering oscillation cues, braking-induced vibration signatures, and tire surface degradation proxies (Figure 1).

**Figure 2:**
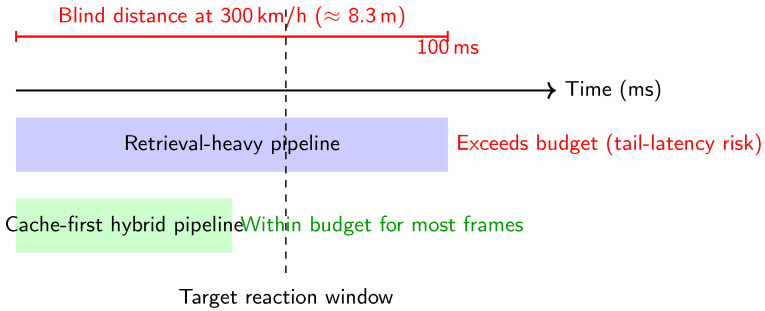
Latency budget motivation. Retrieval-heavy perception can exceed a practical reaction window at racing speeds. A cache-first design aims to keep most frames within a sub-50 ms operational envelope and reduce tail-latency excursions.

The remaining barrier is **deterministic tail latency**. At 300 km/h (≈83.3 m/s), a 100 ms end-to-end delay implies an 8.3 m “blind distance”, which is incompatible with safety-relevant monitoring unless most frames are processed within a tight and predictable budget. While edge accelerators enable local video inference, similarity retrieval can introduce additional (and often non-deterministic) overhead due to vector search and re-ranking [10,12]. This motivates a design that minimizes expensive retrieval calls and explicitly targets *tail-latency* stability rather than optimizing only average throughput. Figure 2 summarizes this operational gap.

**Figure 3:**
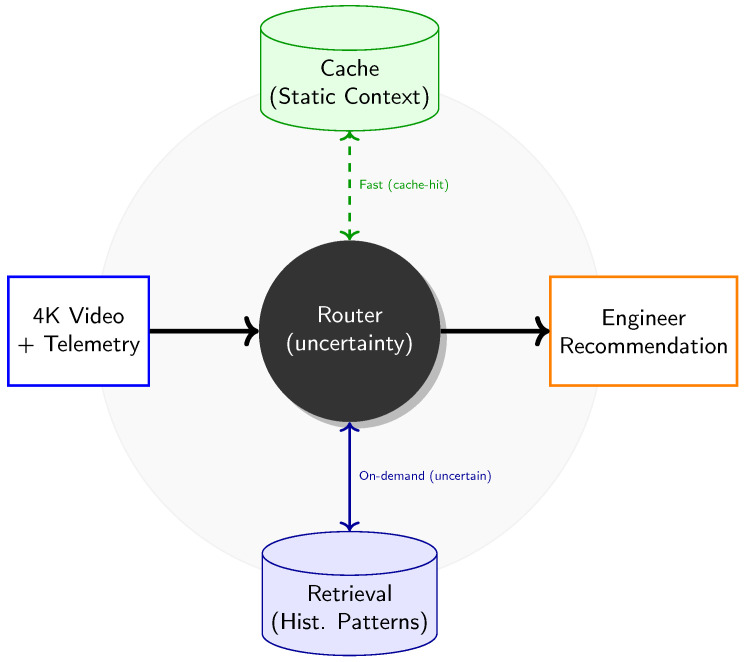
System concept. An uncertainty-driven router prioritizes a cache-first static context path for nominal lapping and triggers historical similarity retrieval only when anomaly/uncertainty signals justify the cost.

To address this, we propose a **hybrid cache–retrieval inference framework** (Figure 3) that routes perception through two memory paths: a *static cache* for circuit-level context and an *on-demand local similarity retrieval* channel for uncertainty-triggered anomaly grounding. Importantly, retrieval is used primarily to *ground ambiguous frames*, improve interpretability, and stabilize decisions under high uncertainty (rather than acting as an additional classifier). Routing is governed by uncertainty/entropy signals: low-entropy frames follow the cache-only path, whereas high-entropy frames trigger retrieval and selective refinement. For implementation, we adopt a lightweight deliberation-and-action loop (ReAct-style) [13] as a routing pattern, while fine-grained texture extraction uses **UNet++** to preserve high-frequency cues [14]. Efficient similarity search follows standard practice (e.g., FAISS indexing) [12], and our uncertainty design is informed by modern guidance beyond raw softmax entropy [15,16].

For clarity of scope, the evaluation in this work assumes a **closed-circuit** setting with synchronized onboard video and telemetry; the camera configuration (mounting position, FOV, orientation, and stabilization assumptions) is specified explicitly in Section 3. The system is intended for **passive monitoring and decision support**, producing advisory outputs without actuating ECU control strategies.

This paper makes the following contributions:

1. **Hybrid cache–retrieval inference for strict latency budgets:** We introduce a cache-first pathway for O(1) access to static circuit context and an on-demand retrieval pathway for rare, uncertain events, explicitly targeting reduced tail-latency excursions on edge hardware.
2. **Uncertainty-guided routing policy:** We propose an entropy/uncertainty-driven router that minimizes unnecessary retrieval calls and improves decision stability at safety-oriented operating points.
3. **Texture-sensitive encoder with selective refinement:** We combine a lightweight backbone with UNet++ refinement to preserve high-frequency texture cues relevant to tire degradation and vibration signatures [10,14].
4. **Motorsport benchmark and evaluation under controlled anomaly injections:** We validate the approach on synchronized telemetry–video data with controlled anomaly injections and report both detection performance and end-to-end latency behavior under realistic constraints.

The remainder of this paper is organized as follows. Section 2 reviews related work on latency-aware video analytics, anomaly detection, and hybrid cache–retrieval strategies. Section 3 describes the proposed architecture and routing policy, including the explicit camera specification. Section 4 details the dataset, anomaly injection protocol, baselines, and evaluation metrics. Section 5 reports quantitative results, including latency distributions and detection performance. Section 6 discusses deployment considerations and limitations, including the intended role as a **passive monitoring and decision-support** module. Finally, Section 7 concludes the paper and outlines future work.

For a detailed visual analysis of how the 2027 technical regulations—specifically the reduction in engine displacement and the prohibition of ride-height devices—alter the vehicle dynamics envelope, please refer to the motivation schematics in Appendix A.

## 2. Related Work

**Figure 4:**
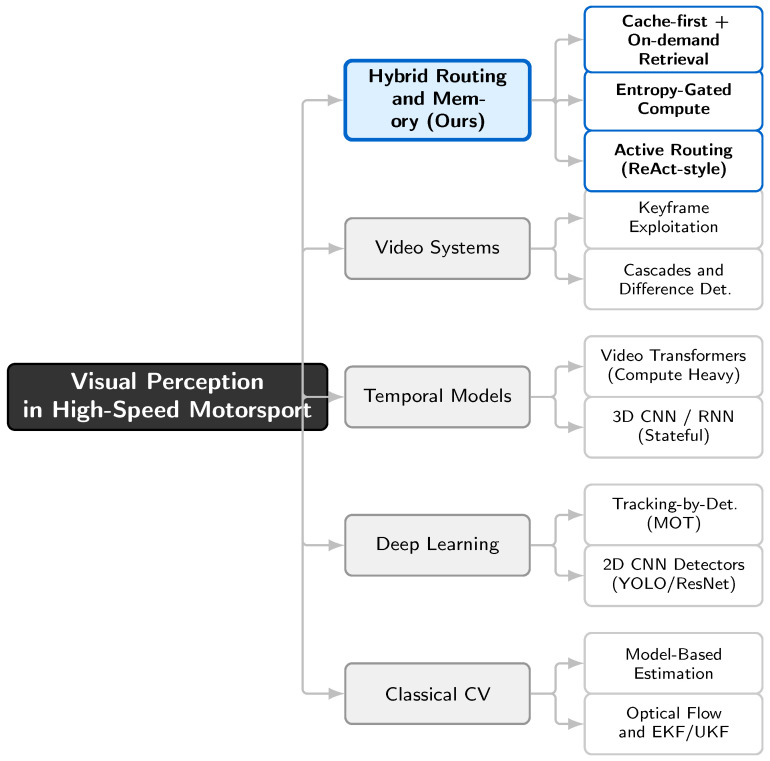
Taxonomy of real-time perception in motorsport. Representative strands include latency-limited high-speed perception [18], motion-artifact-aware imaging [19,20,21], autonomous racing platforms [22,23], cascade-based video specialization [24], dynamic–conditional inference [25,26,27], and memory augmentation via retrieval–caching [17,28,29,30,31]. Our approach integrates (i) uncertainty-gated conditional computation and (ii) a *hybrid cache–retrieval memory* designed for tight latency budgets and bounded tail behavior.

We position our contribution at the intersection of (i) high-speed visual perception under strict latency constraints, (ii) conditional computation and routing for budgeted inference, and (iii) memory-augmented inference that exploits the spatiotemporal redundancy of closed-circuit motorsport. Following reviewer guidance, we use hybrid cache–retrieval inference as the primary framing throughout: a cache-first path handles nominal frames, while on-demand similarity retrieval is reserved for genuinely uncertain or novel events. (In the LLM literature, “Retrieval-Augmented Generation (RAG)” refers to retrieval-augmented text generation [17]. In this paper, retrieval augments visual–telemetry inference and is used to ground ambiguous frames and stabilize decisions; no language generation is performed.) Figure 4 summarizes major paradigms and highlights the gap addressed by this work.

### 2.1. High-Speed Vision Under Hard Latency Constraints

High-speed motorsport perception differs from conventional driving due to extreme ego-motion, vibration, motion blur, and rolling-shutter distortions, as Table 1. In robotics, the impact of perception latency on safe speed has been formalized and shown to be a fundamental limiting factor [18]. From a sensing perspective, event cameras mitigate motion blur and provide microsecond temporal resolution [19], while rolling-shutter distortion in frame cameras can be significant under aggressive motion, motivating both modeling and learning-based corrections [20,21]. Autonomous racing platforms such as F1TENTH further reinforce that perception must be both fast and reliable under strict real-time constraints [22,23].

### 2.2. Conditional Computation and Uncertainty-Triggered Escalation

A recurring systems principle is to reserve expensive computation for rare, ambiguous frames. Dynamic/conditional inference adapts network depth or execution per input to trade computation for accuracy [25,26,27]. However, a key practical issue is that naive confidence measures can be miscalibrated [33]. This motivates uncertainty-aware escalation mechanisms, where higher-cost analysis is triggered only when uncertainty or novelty is detected, and uncertainty estimation is designed beyond raw softmax scores [15,16]. Agentic formulations (e.g., ReAct-style patterns) provide an implementation lens for such routing decisions in a modular pipeline [13].

### 2.3. Redundancy Exploitation in Video Analytics

Closed-circuit racing exhibits strong spatiotemporal redundancy: background geometry and many landmarks remain quasi-stationary across laps. Systems work on video analytics exploited this property via specialization and cascades. NoScope, for example, demonstrated large savings by specializing to a fixed distribution and using lightweight triggers to avoid expensive inference on redundant frames [34]. Translating this insight to motorsport suggests a cache-first path for nominal lapping, with escalation mechanisms reserved for novelty conditions.

### 2.4. Memory-Augmented Inference: Cache Versus Retrieval

Memory augmentation couples a parametric model with non-parametric memory accessed at inference time. In real-time settings, the dominant overhead is approximate nearest-neighbor search over embeddings, commonly implemented with FAISS [28] or graph indices such as HNSW [29]. Recent work on cache augmentation emphasizes that when context is invariant, preloading and reusing representations can remove memory access from the critical path for most inputs [30]. For vision, multimodal retrieval methods operate directly in embedding space to provide historical exemplars [31]. In our setting, retrieval is primarily useful to *ground* ambiguous frames with evidence, thereby improving interpretability and stabilizing decisions, while caching should dominate whenever lap-to-lap context is repetitive.

### 2.5. Summary of the Gap

Prior work has advanced high-speed imaging robustness [18,19,20], conditional inference [25,26], and memory augmentation [28,29,30,31], but these components are often studied in isolation. Motorsport-grade deployment under strict timing constraints motivates their integration into a *routing controller* [13] that (i) maximizes cache hits under nominal lapping and (ii) escalates to on-demand retrieval only when uncertainty/novelty signals justify the cost, explicitly targeting bounded tail-latency behavior.

## 3. Methodology

### 3.1. Problem Setting and Real-Time Constraints

We address real-time visual anomaly monitoring in high-speed motorsport as a stream-to-decision task under strict latency and energy constraints [9,18]. Let ${\left\{\left(I_{t},S_{t}\right)\right\}}_{t\geq 1}$ be synchronized observations [8], where $I_{t}\in R^{H\times W\times 3}$ is an RGB frame and $S_{t}\in R^{d_{s}}$ a telemetry packet (e.g., IMU, suspension travel, wheel speeds, throttle/brake).

At each time step, the system outputs (i) an anomaly posterior and (ii) an advisory engineering vector [11]:

(1) $$a_{t}\;=\;F\left(I_{t},S_{t}\right)\;\in \;R^{K+q},$$

where the first *K* components encode the contextual posterior $p_{t}^{\mathrm{ctx}}\left(c\right)=p\left(y_{t}=c|I_{t},S_{t}\right)$ over anomaly classes, and the remaining *q* components encode advisory outputs (e.g., alert level, recommended data capture, suggested setup check). We explicitly restrict the framework to passive monitoring and decision support (no actuation/ECU control claims).

Camera Acquisition Model (Explicit, Consolidated)

To avoid implicit assumptions, we define a fixed camera model used throughout the evaluation. We assume a rigidly-mounted onboard perspective camera with constant intrinsics and a fixed pose across laps (engineering mount), oriented forward-facing approximately aligned with the vehicle longitudinal axis, with a wide field-of-view suitable for on-vehicle mounting. No electronic stabilization is assumed; instead, vibration/motion blur and rolling-shutter effects are modeled as bounded perturbations in the data generation pipeline (see Section 4.3). This consistency assumption is required for reliable circuit redundancy exploitation (cache sectoring), while the controlled perturbations stress robustness to motion artifacts.

Pipeline Factorization (Modules and Interfaces)

We model F as a composition of explicit modules:

(2) $$F\;=\;\pi_{\mathrm{dec}}\circ \Psi \circ M_{g_{t}}\circ G_{\phi }\circ \Phi \circ E_{\theta }\circ P,$$

where P preprocesses frames (decode/resize/normalize), $E_{\theta }$ is the visual encoder [14,35], Φ normalizes and aligns telemetry [8], $G_{\phi }$ performs budget-aware routing (uncertainty/novelty-driven) [25,26], $M_{g_{t}}$ is the **hybrid cache–retrieval inference** memory interaction (fast cache versus similarity retrieval) [30], Ψ fuses visual–telemetry–memory context into a joint state, and $\pi_{\mathrm{dec}}$ maps this state to $a_{t}$ [13]. **Terminology note:** although the LLM literature often uses the term “RAG” for retrieval-augmented pipelines [17], our system *does not perform text generation*. We therefore use **hybrid cache–retrieval inference** as the primary term, and use “RAG” only when referencing prior literature or analogies.

Fast Posterior versus Contextual Posterior (Routing Clarity)

We distinguish a lightweight *fast* posterior used only for routing from the final contextual posterior used for reporting:

$$p_{t}^{\mathrm{fast}}={\mathrm{Head}}_{\mathrm{fast}}\left(v_{t},s_{t}\right),\;p_{t}^{\mathrm{ctx}}={\mathrm{Head}}_{\mathrm{ctx}}\left(z_{t}\right),$$

where $v_{t}=E_{\theta }\left(P\left(I_{t}\right)\right)$, $s_{t}=\Phi \left(S_{t}\right)$, and $z_{t}=\Psi \left(v_{t},s_{t},c_{t}\right)$ after memory context $c_{t}$ is selected/constructed. Entropy/novelty signals are computed from $p_{t}^{\mathrm{fast}}$ (cheap, stable), while evaluation uses $p_{t}^{\mathrm{ctx}}$.

Objective Under Hard Constraints

Design is guided by constrained risk minimization [25]:

(3) $$\min_{\theta }\;\;E\left[L_{\mathrm{task}}\left(y_{t},{\hat{y}}_{t}\right)\right]\;+\;\lambda \;E\left[L_{\mathrm{cal}}\left(p_{t}^{\mathrm{fast}}\right)\right]\;s.t.\;P\;\left(L_{\mathrm{total}}\left(t\right)>B\right)\leq \alpha ,\;\;E\left[E_{t}\right]\leq \bar{E},$$

where $L_{\mathrm{task}}$ is cross-entropy/focal loss, $L_{\mathrm{cal}}$ is a calibration penalty stabilizing entropy/novelty routing [33], B is the real-time deadline, α is a tail-latency violation probability (chance constraint), and $\bar{E}$ is an energy budget [10]. Table 2 summarizes the key notation and constraints used throughout this formulation. While standard metrics emphasize overall accuracy, our safety-relevant setting also reports recall at high-precision tiers to reduce missed hazardous anomalies.

Latency Budget and Blind Distance

At speed $v_{t}$ (m/s), end-to-end latency $L_{\mathrm{total}}\left(t\right)$ implies blind distance $D_{\mathrm{blind}}\left(t\right)=v_{t}\cdot L_{\mathrm{total}}\left(t\right)$ [18]. Given a safety margin $D_{\max}$ (e.g., braking-marker tolerance), the constraint can be stated as

(4) $$D_{\mathrm{blind}}\left(t\right)\leq D_{\max}\;⟺\;L_{\mathrm{total}}\left(t\right)\leq B\left(t\right)=\frac{D_{\max}}{v_{t}}.$$

In practice, we enforce a conservative constant deadline $B=\min_{t}B\left(t\right)$ over the target operating envelope [10].

Latency Decomposition and the Dominant Memory Term

We decompose end-to-end latency into measurable components [10]:

(5) $$L_{\mathrm{total}}\left(t\right)\;=\;L_{\mathrm{pre}}\left(t\right)+L_{\mathrm{enc}}\left(t\right)+L_{\mathrm{gate}}\left(t\right)+L_{\mathrm{mem}}\left(t\right)+L_{\mathrm{post}}\left(t\right)\;\;\leq \;\;B,$$

where $L_{\mathrm{post}}\left(t\right)=L_{\mathrm{fuse}}\left(t\right)+L_{\mathrm{dec}}\left(t\right)$. The variable term $L_{\mathrm{mem}}\left(t\right)$ dominates whenever similarity retrieval is invoked [28]. We model it explicitly using a gate $g_{t}\in \left\{0,1\right\}$ [25]:

(6) $$L_{\mathrm{mem}}\left(t\right)=\left(1-g_{t}\right)\;L_{\mathrm{CAG}}\;+\;g_{t}\;L_{\mathrm{RET}},$$

where $L_{\mathrm{CAG}}$ is approximately constant-time (cache lookup) [30], while $L_{\mathrm{RET}}$ includes ANN search and context assembly [29].

Tail-Latency (Safety-Relevant)

Average-case compliance is insufficient; we track percentiles and enforce a chance constraint [10,18]:

(7) $$P\;\left(L_{\mathrm{total}}\left(t\right)>B\right)\leq \alpha ,\;\mathrm{equivalently}\;p_{\left(1-\alpha \right)}\;\left(L_{\mathrm{total}}\right)\leq B,$$

where $p_{\left(1-\alpha \right)}$ denotes the (1−α) latency percentile (e.g., p99 for α=0.01). This is crucial because ANN retrieval can be data-dependent and exhibit tail behavior even when expected time is small [28,29]. The breakdown of these latency components is detailed in Table 3.

Energy Proxy and Expected Energy Under Routing

On edge platforms, energy correlates with module time and power [10,36]:

(8) $$E_{t}=\sum_{i}P_{i}\;L_{i}\left(t\right),$$

where $P_{i}$ is effective power draw of module *i* during execution. Using the same mixture form as Equation (6), expected energy admits

(9) $$E\left[E_{t}\right]={\bar{E}}_{\setminus \mathrm{mem}}+E_{\mathrm{CAG}}+\mathrm{Pr}\left(g_{t}=1\right)\left(E_{\mathrm{RET}}-E_{\mathrm{CAG}}\right),$$

making explicit that reducing the retrieval rate $\mathrm{Pr}(g_{t}=1)$ reduces expected latency and energy [25,26].

### 3.2. System Overview

**Figure 5:**
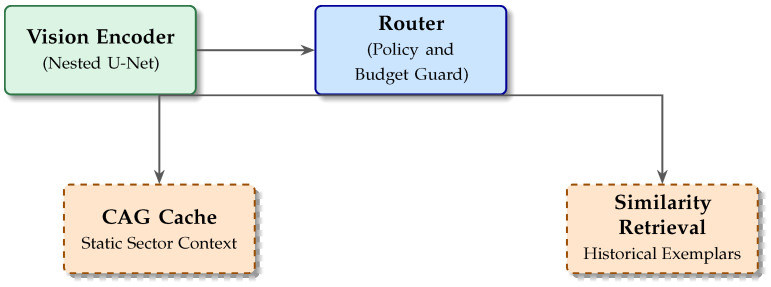
High-level module interaction. The encoder produces embeddings $v_{t}$; a budget-aware router selects either a fast cache path (CAG) for invariant circuit context or a similarity retrieval path (ANN) to ground uncertain events in historical exemplars.

The proposed framework is a heterogeneous, budget-aware perception stack that minimizes *expected* inference latency while preserving semantic depth for *interpretable* anomaly assessment under safety-relevant operating points [25]. Figure 5 summarizes the module interaction.

Hybrid Cache–Retrieval Inference as a Two-Expert Policy

We interpret the system as a two-expert mixture (fast cache expert versus deep similarity-retrieval expert) with a budget-aware gate [25,27]. Let $U_{t}$ denote an uncertainty/novelty score (Section 3.4.2) and let ${\hat{L}}_{\mathrm{CAG}}$ and ${\hat{L}}_{\mathrm{RET}}^{\left(p99\right)}$ denote online latency estimates. The routing decision is implemented as a deterministic policy with stability constraints (EMA + hysteresis + dwell time) to ensure bounded tail latency.

Role of Retrieval (Reviewer-Critical Distinction)

Similarity retrieval primarily improves **interpretability and decision stability**: it grounds uncertain detections in *retrieved historical exemplars* (metadata + nearest-neighbor context) and reduces mode-flips under ambiguity (via contextual consistency), rather than acting as a generic “accuracy booster.” Classification improvements can occur, but the primary methodological role is to stabilize the advisory decision state $z_{t}$ and provide engineer-auditable evidence trails.

Role of Retrieval (Reviewer-Critical Distinction)

Similarity retrieval primarily improves interpretability and decision stability: it grounds uncertain detections in retrieved historical exemplars (metadata + nearest-neighbor context) and reduces mode-flips under ambiguity (via contextual consistency), rather than acting as a generic “accuracy booster.” Classification improvements can occur, but the primary methodological role is to stabilize the advisory decision state $z_{t}$ and provide engineer-auditable evidence trails. Table 4 summarizes the architectural components and their latency criticality.

### 3.3. Hierarchical Feature Extraction (Nested U-Net)

This subsection describes the hardware-aware encoder that converts high-rate video into a retrieval-friendly embedding while preserving fine-grained texture and micro-oscillation cues indicative of early instability.

Hardware-Aware Preprocessing (Zero-Copy)

Raw 4K frames $I_{t}\in R^{3840\times 2160\times 3}$ are downsampled and center-cropped to ${\tilde{I}}_{t}\in R^{512\times 512\times 3}$ via a zero-copy hardware path to avoid CPU bottlenecks and reduce memory bandwidth on the GPU critical path [10]:

(10) $${\tilde{I}}_{t}=P\left(I_{t}\right),\;P:R^{3840\times 2160\times 3}\;\to \;R^{512\times 512\times 3}.$$

To stabilize inference under illumination changes, we apply lightweight per-frame affine normalization:

(11) $${\hat{I}}_{t}=\mathrm{clip}\;\left(\frac{{\tilde{I}}_{t}-\mu_{t}}{\sigma_{t}+\epsilon }\right),$$

where $\mu_{t},\sigma_{t}$ are per-channel statistics (computed on-GPU), and ϵ prevents division by zero.

Encoder Definition (Embedding + Optional Cues)

The encoder $E_{\theta }$ maps normalized inputs to a compact embedding:

(12) $$v_{t}=E_{\theta }\left({\hat{I}}_{t}\right)\in R^{d_{v}},\;d_{v}=512,$$

and optionally to dense cue maps $Q_{t}$ used for interpretability and offline diagnostics (not required on the real-time path):

(13) $$Q_{t}=h_{\mathrm{cue}}\left(\left\{\;x^{i,j}\;\right\}\right)\in R^{H^{\prime }\times W^{\prime }\times C_{q}}.$$

**Figure 6:**
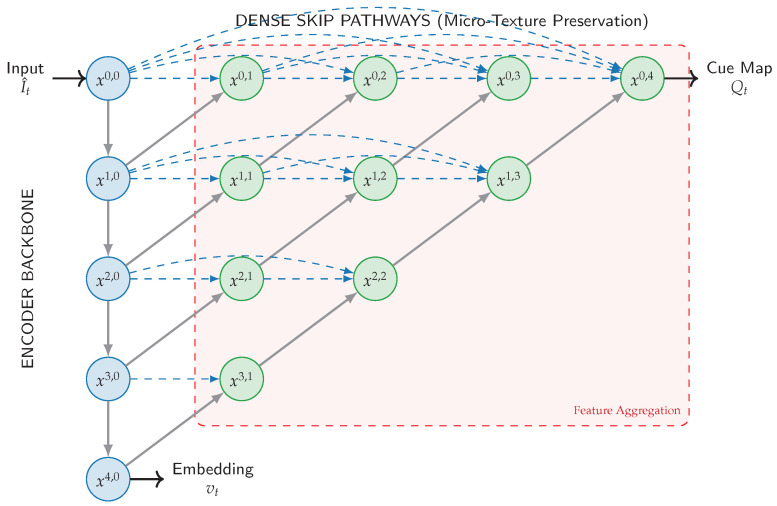
Nested U-Net Architecture. The nested topology aggregates multi-scale features via dense skip connections, preserving high-frequency cues (e.g., chatter) that may vanish in deep bottlenecks.

We implement a UNet++-style nested skip aggregation with a lightweight backbone to preserve high-frequency texture cues while remaining edge-feasible [14,37], as illustrated in Figure 6.

UNet++ Aggregation (Formal)

Let $x^{i,j}\in R^{H_{i}\times W_{i}\times C_{i}}$ denote the feature map at depth *i* and nested stage *j*. The aggregation is [14]

(14) $$x^{i,j}=\left\{\begin{array}{cc} H^{i,0}\left(x^{i-1,0}\right), & j=0,\\ H^{i,j}\;\left(\left[x^{i,0},x^{i,1},\ldots ,x^{i,j-1},\;U\left(x^{i+1,j-1}\right)\right]\right), & j>0, \end{array}\right.$$

where $H^{i,j}$ is a Conv–Norm–Nonlinearity block, U is upsampling, and [·] is channel concatenation.

Embedding Normalization for Retrieval

To make embeddings comparable under cosine similarity, we apply $L_{2}$ normalization:

(15) $${\tilde{v}}_{t}=\frac{v_{t}}{∥v_{t}{∥}_{2}+\epsilon }.$$

Composite Loss (Classification + Cues + Retrieval Geometry)

To ensure embeddings are retrieval-friendly and class-discriminative, we use

(16) $$L=\alpha \;L_{\mathrm{cls}}+\beta \;L_{\mathrm{cue}}+\gamma \;L_{\mathrm{retr}}.$$

Classification loss. Weighted cross-entropy or focal loss:

(17) $$L_{\mathrm{cls}}=-\sum_{k=1}^{K}w_{k}\;{\left(1-p_{t}\left(k\right)\right)}^{\eta }\;I\left[y_{t}=k\right]\log p_{t}\left(k\right).$$

Cue loss. Dice loss:

(18) $$L_{\mathrm{cue}}=1-\frac{2\sum_{u}{\hat{q}}_{u}q_{u}+\epsilon }{\sum_{u}{\hat{q}}_{u}^{2}+\sum_{u}q_{u}^{2}+\epsilon }.$$

Retrieval loss. Supervised contrastive loss [38]:

(19) $$L_{\mathrm{retr}}=\sum_{i\in B}\frac{-1}{\left|P(i)\right|}\sum_{p\in P(i)}\log\frac{\exp({\tilde{v}}_{i}^{⊤}{\tilde{v}}_{p}/\tau )}{\sum_{a\in A(i)}\exp\left({\tilde{v}}_{i}^{⊤}{\tilde{v}}_{a}/\tau \right)},$$

where P(i) are positives sharing the same anomaly label (or same failure mode/sector). Table 5 details the specific architecture configuration and training hyperparameters.

### 3.4. Budget-Aware Orchestration and Uncertainty

**Figure 7:**
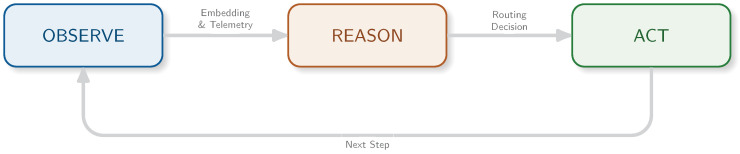
Budget-aware ReAct loop. The agent observes the fused state, reasons via calibrated uncertainty/novelty signals, and acts by selecting the compute path (cache versus retrieval) under the deadline constraint.

**Figure 8:**
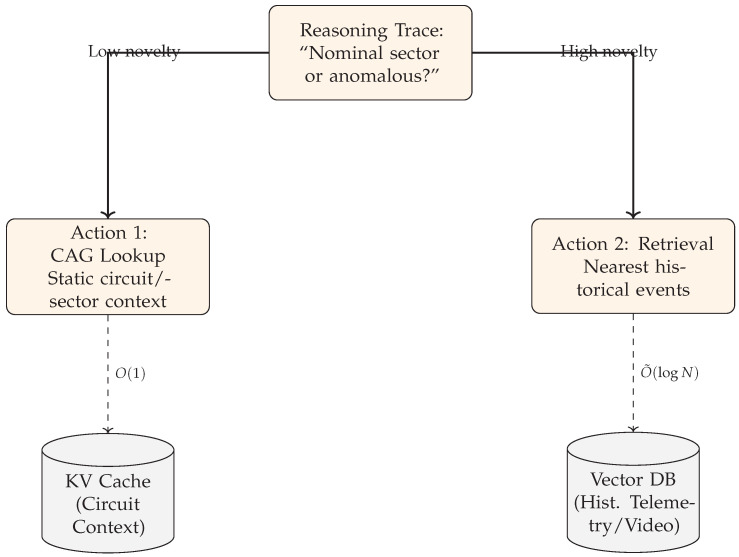
Specialized routing. The agent uses CAG for static invariants and escalates to similarity retrieval only when calibrated novelty indicates uncertainty under the time budget.

The orchestrator implements an Observe–Reason–Act loop specialized to tool routing under a hard deadline [13], as illustrated in Figure 7. It decides when to use the fast cache (CAG) versus similarity retrieval (ANN over historical exemplars), while enforcing stability constraints (EMA + hysteresis + dwell time) to reduce thrashing and tail-latency variance [25]. The routing logic is depicted in Figure 8.

State Fusion

We define fused observation $o_{t}=\left(v_{t},s_{t}\right)$, where $s_{t}=\Phi \left(S_{t}\right)$ is normalized telemetry [8]:

(20) $$s_{t}=\Phi \left(S_{t}\right),\;o_{t}=\left(v_{t},s_{t}\right).$$

#### 3.4.1. Uncertainty Quantification and Calibration

Calibrated Predictive Entropy (Routing Signal)

We derive uncertainty from the predictive distribution $p_{t}^{\mathrm{fast}}$. Let $\ell_{t}^{\mathrm{fast}}\in R^{K}$ be fast logits. We apply temperature scaling [33]:

(21) $$p_{t}^{\left(T\right)}=\mathrm{softmax}\;\left(\frac{\ell_{t}^{\mathrm{fast}}}{T_{\mathrm{cal}}}\right),$$

with $T_{\mathrm{cal}}$ fitted on a calibration set by minimizing NLL. The normalized Shannon entropy is

(22) $$H_{t}=\frac{-\sum_{k=1}^{K}p_{t,k}^{\left(T\right)}\log\left(p_{t,k}^{\left(T\right)}+\epsilon \right)}{\log K}\in \left[0,1\right].$$

Calibration Quality (ECE)

We report expected calibration error (ECE) [39]:

(23) $$\mathrm{ECE}=\sum_{m=1}^{M}\frac{|B_{m}|}{n}\left|\mathrm{acc}\left(B_{m}\right)-\mathrm{conf}\left(B_{m}\right)\right|.$$

#### 3.4.2. Novelty and OOD Detection Signals (Multi-Signal Gate)

Entropy alone can be insufficient under distribution shift. We define a composite novelty score used consistently for routing:

(24) $$U_{t}\;=\;\omega_{1}\;H_{t}\;+\;\omega_{2}\;{\tilde{S}}_{t}\;+\;\omega_{3}\;{\tilde{\Delta }}_{t},\;\sum_{i}\omega_{i}=1,\;\omega_{i}\geq 0,$$

where (i) $H_{t}$ is calibrated entropy (Equation (22)), (ii) ${\tilde{S}}_{t}$ is a normalized energy-based OOD score, and (iii) ${\tilde{\Delta }}_{t}$ is a normalized embedding-drift signal.

Energy-Based OOD Score (Normalized)

We compute an energy score from fast logits [20]:

(25) $$E_{t}\;=\;-T_{\mathrm{cal}}\log\sum_{k=1}^{K}\exp\;\left(\frac{\ell_{t,k}^{\mathrm{fast}}}{T_{\mathrm{cal}}}\right).$$

Lower energy indicates in-distribution confidence; to make the term increase with novelty, we use a calibrated normalization:

(26) $${\tilde{S}}_{t}=\mathrm{norm}\left(-E_{t}\right)\in \left[0,1\right].$$

Embedding Drift (Fast Novelty Trigger)
  (27) $$\Delta_{t}={∥{\tilde{v}}_{t}-{\tilde{v}}_{t-1}∥}_{2},\;{\tilde{\Delta }}_{t}=\mathrm{norm}\left(\Delta_{t}\right)\in \left[0,1\right].$$

#### 3.4.3. Stable Tool Routing: Hysteresis + Dwell Time + EMA

**Figure 9:**
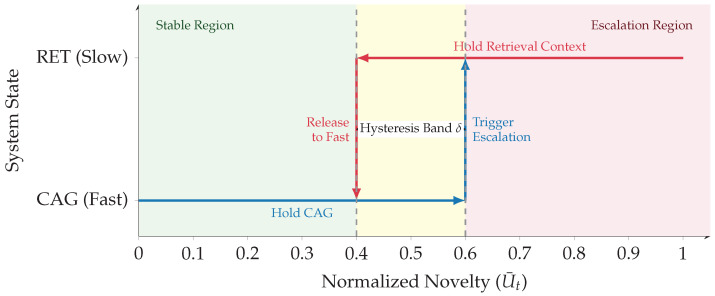
Hysteresis-based routing. The system uses a Schmitt-trigger mechanism to avoid flicker: escalation requires ${\bar{U}}_{t}>\tau_{\mathrm{high}}$, and release requires ${\bar{U}}_{t}<\tau_{\mathrm{low}}$.

To prevent thrashing and ensure operational stability, we implement a hysteretic switching mechanism illustrated in Figure 9. The specific hyperparameters for this logic are detailed in Table 6, and the complete routing process is formalized in Algorithm 1.

EMA Smoothing
  (28) $${\bar{U}}_{t}=\rho \;{\bar{U}}_{t-1}+\left(1-\rho \right)\;U_{t},\;\rho \in \left[0,1\right).$$

Hysteretic Switching (Anti-Flicker)
  (29) $${\mathrm{Mode}}_{t}=\left\{\begin{array}{cc} \mathrm{CAG}, & {\bar{U}}_{t}<\lambda -\delta ,\\ \mathrm{RET}, & {\bar{U}}_{t}>\lambda +\delta ,\\ {\mathrm{Mode}}_{t-1}, & \mathrm{otherwise}. \end{array}\right.$$

Dwell-Time Constraint (Anti-Thrashing)
  (30) $$\mathrm{switch}\;\mathrm{allowed}\;\mathrm{at}\;t\;\Rightarrow \;t-t_{\mathrm{last}\_\mathrm{switch}}\geq m.$$

Hard Deadline Guard (p99-Safe)

To prevent rare ANN slowdowns from violating safety margins, we forbid retrieval when remaining slack is insufficient:

(31) $$g_{t}\leftarrow 0\;\mathrm{if}\;L_{\mathrm{elapsed}}\left(t\right)+{\hat{L}}_{\mathrm{RET}}^{\left(p99\right)}+{\hat{L}}_{\mathrm{post}}^{\left(p99\right)}>B.$$

**Algorithm 1:** Stable, deadline-aware routing (CAG versus retrieval) using composite novelty
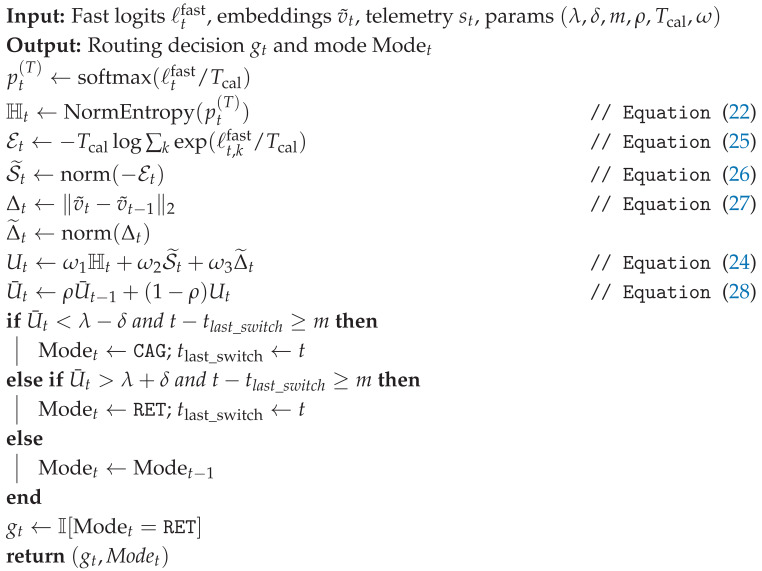


### 3.5. Hybrid Memory Architecture: CAG + Similarity Retrieval

We implement a dual memory system that exploits the spatiotemporal redundancy of closed-circuit racing while keeping tail latency bounded. The two memory banks are

(32) $$M_{\mathrm{CAG}}:\;k\mapsto c_{k}\;(\mathrm{static}\;\mathrm{cache}),\;D_{\mathrm{RET}}={\left\{\left(v_{i},m_{i}\right)\right\}}_{i=1}^{N}\;(\mathrm{similarity}-\mathrm{retrieval}\;\mathrm{store}).$$

Here, $c_{k}$ is a cached context object (sector priors, landmarks, canonical background embeddings, and precomputed reference features), and $\left(v_{i},m_{i}\right)$ are historical exemplars with embedding $v_{i}\in R^{d_{v}}$ and metadata $m_{i}$ (bike spec, tire compound, track, weather, session type, etc.).

#### 3.5.1. Tier 1: Cache-Augmented Inference (CAG)

Keying by Curvilinear Lap Coordinate

CAG stores static circuit context keyed by a curvilinear coordinate $\sigma_{t}\in \left[0,L_{\mathrm{lap}}\right)$ (arc-length along the centerline). We discretize

(33) $$k_{\mathrm{geo}}=\left\lfloor \frac{\sigma_{t}}{\Delta \sigma }\right\rfloor .$$

Estimating $\sigma_{t}$ (Sensor Fusion/Map Matching)

$\sigma_{t}$ can be obtained by map-matching GNSS to a track centerline [40] or by dead-reckoning with periodic correction [23]:

(34) $$\sigma_{t}\;=\;\left(\sigma_{t-1}+{\hat{v}}_{t}^{\|}\;\Delta t\right)\;\mathrm{mod}\;L_{\mathrm{lap}},\;{\hat{v}}_{t}^{\|}=\kappa_{t}\;v_{t}^{\mathrm{wheel}}.$$

Choosing Cache Granularity Δσ

A defensible choice ties spatial resolution to the maximum distance traveled within the perception budget:

(35) $$\Delta \sigma \;\geq \;v_{\max}\;B.$$

O(1) VRAM Lookup

We implement $M_{\mathrm{CAG}}$ as a GPU-resident array/hash indexed by $k_{\mathrm{geo}}$, yielding constant-time access [30]:

(36) $$c_{t}\leftarrow M_{\mathrm{CAG}}\left[k_{\mathrm{geo}}\right],\;O\left(1\right).$$

Prototype Drift Detection (Mahalanobis)
  (37) $$d_{t}^{\mathrm{CAG}}={\left(v_{t}-\mu_{k_{\mathrm{geo}}}\right)}^{⊤}\Sigma_{k_{\mathrm{geo}}}^{-1}\left(v_{t}-\mu_{k_{\mathrm{geo}}}\right).$$

Update versus Spawn Policy

Let ${\bar{d}}_{t}^{\mathrm{CAG}}$ be an EMA of $d_{t}^{\mathrm{CAG}}$:

(38) $${\bar{d}}_{t}^{\mathrm{CAG}}=\rho {\bar{d}}_{t-1}^{\mathrm{CAG}}+\left(1-\rho \right)d_{t}^{\mathrm{CAG}}.$$

If ${\bar{d}}_{t}^{\mathrm{CAG}}$ exceeds a threshold for *w* consecutive frames, we spawn a new node; otherwise, we update the existing prototype:

(39) $$\mu_{k}\leftarrow \left(1-\eta \right)\mu_{k}+\eta v_{t},\;\Sigma_{k}\leftarrow \left(1-\eta \right)\Sigma_{k}+\eta \left(v_{t}-\mu_{k}\right){\left(v_{t}-\mu_{k}\right)}^{⊤}.$$

#### 3.5.2. Tier 2: Similarity Retrieval (ANN over Historical Exemplars)

For high-novelty frames, the system retrieves top-*k* nearest historical exemplars from $D_{\mathrm{RET}}$ using an ANN index. We adopt HNSW graphs [29] due to favorable recall/latency trade-offs.

Similarity and Weighted Context Aggregation

Retrieval maximizes cosine similarity:

(40) $$N_{k}\left(v_{t}\right)=\underset{\left(v_{i},m_{i}\right)\in D_{\mathrm{RET}}}{arg topk}\frac{v_{t}^{⊤}v_{i}}{∥v_{t}{∥}_{2}{∥v_{i}∥}_{2}}.$$

We aggregate retrieved contexts via softmax weighting (temperature $\tau_{s}$):

(41) $$w_{j}=\frac{\exp(\mathrm{sim}\left(v_{t},v_{\left(j\right)}\right)/\tau_{s})}{\sum_{r=1}^{k}\exp\left(\mathrm{sim}\left(v_{t},v_{\left(r\right)}\right)/\tau_{s}\right)},\;c_{t}^{\mathrm{RET}}=\sum_{j=1}^{k}w_{j}\;g\left(m_{\left(j\right)}\right).$$

Domain-Aware Filtering

To prevent obsolete matches, we apply metadata predicates (track, weather, regulations, bike spec) as formalized in Algorithm 2:

(42) $$D_{\mathrm{RET}}^{P}=\left\{\left(v_{i},m_{i}\right)\in D_{\mathrm{RET}}:P\left(m_{i}\right)=1\right\}.$$

**Algorithm 2:** Similarity retrieval with domain filtering and aggregation
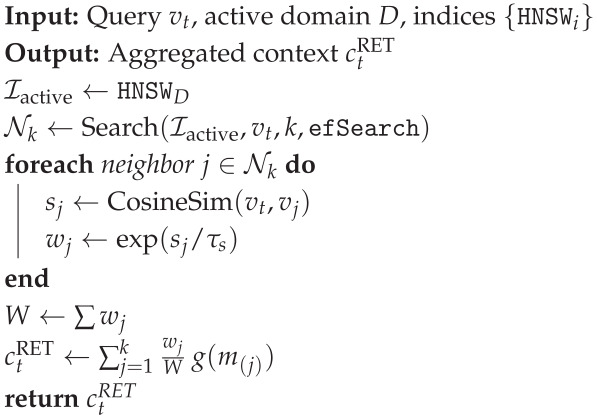

Index Design for Deterministic Latency

To improve p99 stability, we recommend separate indices per domain (and optionally per track):

(43) $${\mathrm{HNSW}}_{\mathrm{track},\mathrm{domain}}\;\Rightarrow \;L_{\mathrm{RET}}\approx L_{\mathrm{ANN}}\left(N_{\mathrm{track},\mathrm{domain}}\right),$$

reducing effective *N* and improving tail behavior [28].

ANN Stack and Reproducibility

On Jetson AGX Orin, we implement retrieval using a GPU-accelerated HNSW index (FAISS) [28]. Hyperparameters are tuned to satisfy the B=50 ms deadline, prioritizing tail-latency stability [29]. Table 7 details the specific memory parameters, while Algorithm 3 outlines the logic for the static context cache maintenance.
**Algorithm 3:** CAG lookup, drift test, and regeneration
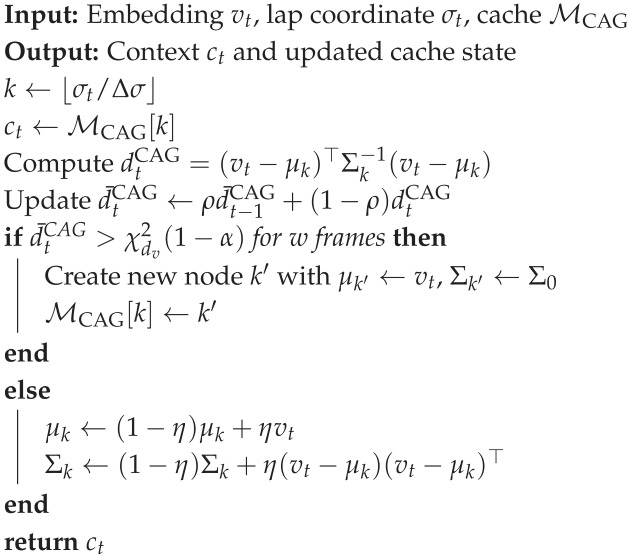


### 3.6. Formal Online Inference Algorithm

Decision-State Fusion

At each time step *t*, we concatenate the current visual embedding, the selected memory context, and normalized telemetry [8]:

(44) $$z_{t}=\left[v_{t}⊕c_{t}⊕s_{t}\right]\in R^{d_{v}+d_{c}+d_{s}},\;a_{t}=\pi_{\mathrm{dec}}\left(z_{t}\right)\in R^{K+q}.$$

We explicitly restrict $a_{t}$ to advisory signals (no actuation claims).

Budget-Aware Streaming Inference (With p99-Safe Guard)

The online pipeline routes to cache versus retrieval using composite novelty $U_{t}$ (Equation (24)), stabilized by EMA + hysteresis + dwell time (Equations (28)–(30)), and protected by the p99-safe guard (Equation (31)). The complete execution flow is presented in Algorithm 4.

Implementation Note (Deterministic Tail Control)

**Figure 10:**
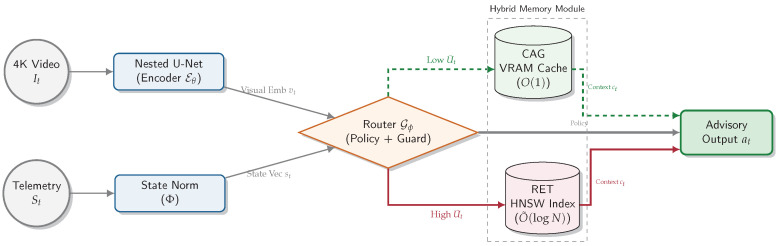
System architecture. Visual embeddings $v_{t}$ and telemetry $s_{t}$ feed a router $G_{\phi }$ that selects a fast VRAM cache (CAG) or a deep ANN similarity-retrieval module (RET) based on composite novelty ${\bar{U}}_{t}$. A p99-safe guard enforces deterministic operation under the 50 ms budget.

To keep p95/p99 latency bounded, we use (i) index partitioning (Equation (43)), (ii) a fixed efSearch cap, and (iii) a retrieval timeout that falls back to CAG if exceeded. This strategy is integral to the overall system architecture depicted in Figure 10, preserving deterministic operation under edge compute variability.
**Algorithm 4:** Budget-aware streaming inference (stable novelty routing + p99-safe deadline guard)
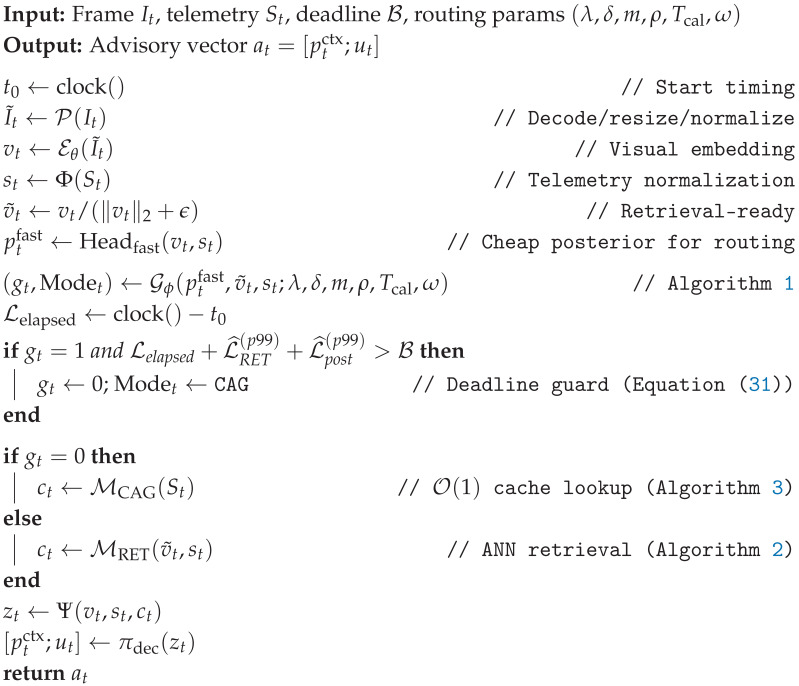


### 3.7. Limitations and Validity Domain (Explicit)

Our claims are intentionally scoped to the evaluated configuration: (i) an **onboard forward-facing wide-FOV camera** with fixed intrinsics/pose across laps (Section 3.1), (ii) a **Jetson AGX Orin** edge target with the latency budget reported in Table 3 and (iii) the **Aspar-Synth-10K** simulation benchmark with controlled domain randomization and injected anomalies (Section 4.3). Performance and latency may differ for alternative camera placements (e.g., helmet/chassis mounts), different sensors (global shutter/event cameras), substantially different tracks, or larger retrieval stores. The hybrid cache–retrieval inference strategy is general, but reported numeric results should not be overgeneralized beyond these operating assumptions without re-calibration and re-profiling of the latency tail.

## 4. Experiments

This section validates the proposed *hybrid cache–retrieval inference* framework under (i) hard real-time constraints with tail-latency control, (ii) embedded power/thermal limits, and (iii) motorsport-inspired operational constraints (edge-only execution and passive monitoring). We evaluate three axes: **(A)** latency distribution and deadline compliance, **(B)** diagnostic quality with safety-critical emphasis on false negatives, and **(C)** energy efficiency under sustained edge operation. In addition, we quantify how retrieval primarily improves *decision stability and interpretability* (grounding) rather than merely inflating classification scores.

### 4.1. Experimental Design, Reproducibility, and Protocol

Design Principles

We enforce (a) **deadline-aware reporting** (P50/P95/P99 latency and deadline-miss rate) following latency–safety analyses in high-speed robotics [18]; (b) **controlled ablations** isolating cache, retrieval, routing stability, and condition filtering; and (c) **edge-only measurement** where all latency and power metrics are captured on the deployment device [10].

Camera Configuration (Explicit, Consolidated)

All experiments assume the same **rigidly-mounted onboard** forward-facing perspective camera model described in Section 4.3: fixed intrinsics and pose across laps (engineering mount), wide field-of-view appropriate for on-vehicle mounting, and no electronic stabilization. Motion artifacts (vibration, blur, rolling shutter) are introduced through controlled simulation perturbations within bounded ranges, ensuring a consistent visual reference for caching while stressing robustness.

Data Splits and Leakage Prevention

To prevent temporal/track-location leakage in memory-augmented systems, we split by *lap index* and *session seed*. All memory stores are built exclusively from training laps: (i) the retrieval bank $D_{\mathrm{RET}}$ is built from training laps only; (ii) cache (CAG) prototypes are populated offline from training laps only; and (iii) evaluation uses *frozen* cache nodes and a *frozen* retrieval bank, ensuring retrieval at time *t* cannot access test data.

Measurement Protocol

All timing is captured with CUDA events around module boundaries consistent with the budgeted routing constraint and the runtime tail guard (Section 3.4). Power is measured from on-device telemetry rails (Section 4.4.2). Algorithm 5 summarizes the benchmark procedure.
**Algorithm 5:** Edge Measurement Protocol (Latency and Power)
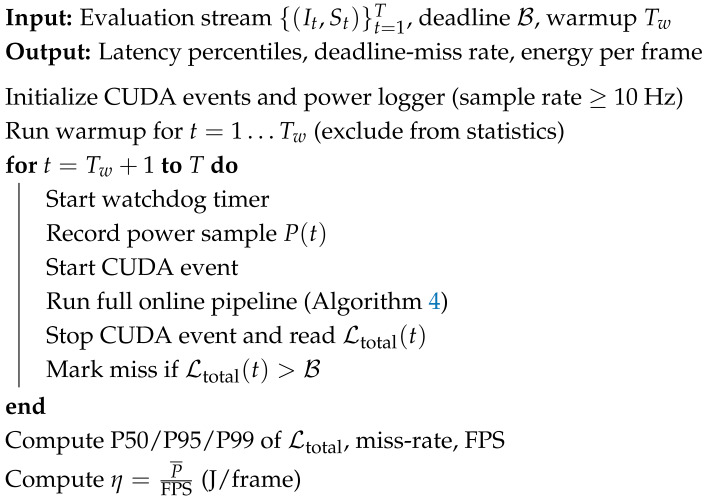


### 4.2. Hypotheses

We investigate three hypotheses aligned with the paper’s core contributions (budgeted routing + hybrid memory):

- **H1 (Latency and Tail Control):** Cache-first routing reduces both average inference time and tail latency compared to retrieval-always baselines, maintaining $L_{\mathrm{total}}<B$ with a low deadline-miss rate.
- **H2 (Safety-Critical Detection and Stability):** On-demand similarity retrieval improves *grounding* of uncertain events, stabilizes per-frame decisions under ambiguity, and reduces false negatives for critical dynamic anomalies (e.g., chatter/headshake), relative to memoryless and cache-only baselines.
- **H3 (Energy Efficiency):** Hybrid routing improves energy efficiency (J/frame and FPS/W) compared to always-on retrieval, enabling sustained operation within embedded thermal constraints.

### 4.3. Simulation Environment and Dataset (Aspar-Synth-10K)

#### 4.3.1. Simulation Environment and Rendering Pipeline

To mitigate safety risks of fault injection during physical testing, we employ a high-fidelity simulation workflow [23]. We generate ***Aspar-Synth-10K***, comprising synchronized 4K onboard video and 100 Hz telemetry streams. The circuit geometry is inspired by an *Aspar*-like layout to validate spatial caching and lap-to-lap repeatability.

Domain Randomization

Following established protocols [26], we randomize (i) illumination/weather (dry, wet, overcast, sunset), (ii) camera vibration/motion blur profiles, and (iii) progressive mechanical degradations. We also randomize **track surface roughness** (micro-bumps) to create hard negatives that can induce visual vibration without mechanical failure (Section 4.8).

#### 4.3.2. Anomaly Taxonomy and Injection Realism

We define *K* anomaly classes spanning (1) suspension oscillations (chatter bands, headshake) [41], (2) braking-induced vibration, (3) tire surface degradation cues (visual texture evolution), (4) curb impact/transient instability, and (5) nominal state. Each anomaly is injected via a parameter schedule controlling frequency, amplitude, onset lap, and persistence. Table 8 provides the specific taxonomy and parameters used in the dataset generation.

Injection Mechanism (Non-Trivial Cues)

To avoid trivial cues, anomalies are generated by physics-parameter perturbations and temporally coherent render changes, not by simple overlays:

- Chatter/headshake: band-limited excitation introduced through suspension/tire dynamics parameters, producing coherent motion/vibration patterns over time.
- Brake resonance: pressure-coupled vibration with onset tied to braking events.
- Tire degradation: progressive texture evolution (accumulative) coupled to stint/lap progression.
- Curb impacts: transients tied to curb-contact events and decaying over a short horizon.

Ground-truth labels are assigned from simulator state and injection schedule, with temporal tolerances to avoid labeling single-frame artifacts.

### 4.4. Hardware and Deployment

#### 4.4.1. Offline Training

Deep model training and cache population are executed offline on a workstation GPU (FP32/FP16 as available). All weights are frozen before edge evaluation for reproducibility.

#### 4.4.2. Edge-Side Inference and Power Logging

All reported latency and power metrics are measured on an embedded deployment target (NVIDIA Jetson AGX Orin). We export the encoder and heads via TensorRT with INT8 quantization and calibrated scaling to ensure deterministic inference and minimize memory footprint [36]. Power is logged from on-device telemetry rails (INA3221) and converted to J/frame via $\eta =\bar{P}/\mathrm{FPS}$ [10]. Table 9 summarizes the configuration.

### 4.5. Baselines and Ablations

To isolate the contribution of each component (memory, routing stability, refinement, and condition filtering), we compare the variants listed in Table 10 (incremental ablation design). We use hybrid cache–retrieval inference as the primary framing; the labels “CAG” (cache) and “RET” (similarity retrieval) are used only as shorthand.

### 4.6. Evaluation Metrics (Safety, Quality, Efficiency)

Real-Time Compliance (Safety Timing)

We characterize the latency distribution of $L_{\mathrm{total}}$ via P50/P95/P99 [18]. The **deadline miss rate (DMR)** is

(45) $$\mathrm{DMR}=\frac{1}{N}\sum_{t=1}^{N}I\;\left[L_{\mathrm{total}}\left(t\right)>B\right],$$

where *N* is the number of evaluated frames, and B=50 ms. We also report **blind distance** $D_{\mathrm{blind}}=v\cdot L$ under representative top-speed *v*.

Diagnostic Quality (Safety-Critical Emphasis)

In addition to **Macro-F1**, we report **per-class recall** and **false negative rate (FNR)** for critical anomalies:

(46) $${\mathrm{FNR}}_{k}=1-{\mathrm{Recall}}_{k},\;k\in \left\{\mathrm{headshake},\mathrm{chatter},\mathrm{brake}\right\}.$$

Routing and Memory Behavior (Interpretability/Stability)

To validate uncertainty-driven routing, we report (i) **escalation recall** ER (fraction of anomaly frames escalated to retrieval), and (ii) **waste rate** WR (fraction of nominal frames escalated):

(47) $$\mathrm{ER}=\frac{\sum_{t}I\left[y_{t}\neq 0\wedge g_{t}=1\right]}{\sum_{t}I\left[y_{t}\neq 0\right]},\;\mathrm{WR}=\frac{\sum_{t}I\left[y_{t}=0\wedge g_{t}=1\right]}{\sum_{t}I\left[y_{t}=0\right]}.$$

We also report retrieval relevance Rel@*k* (fraction of retrieved neighbors matching the GT class family) and calibration metrics (ECE) for routing reliability [39]. Finally, to capture **decision stability**, we report a *flip rate*:

(48) $$\mathrm{FlipRate}=\frac{1}{N-1}\sum_{t=2}^{N}I\;\left[\arg\max p_{t}^{\mathrm{ctx}}\neq \arg\max p_{t-1}^{\mathrm{ctx}}\right],$$

reported separately for nominal and anomalous segments. This metric directly tests whether retrieval primarily stabilizes decisions under ambiguity.

Efficiency and Thermal Viability

We quantify **energy per frame** ($J_{f}$) and throughput (FPS) under the device power cap [10]. We also report the **retrieval escalation rate**:

(49) $$\pi_{\mathrm{RET}}=\frac{1}{N}\sum_{t=1}^{N}g_{t},\;g_{t}\in \left\{0,1\right\}.$$

Statistical Treatment (Confidence Intervals)

We compute **bootstrap 95% confidence intervals** for Macro-F1 and critical-class recall by resampling at the *lap* level. We additionally apply a paired non-parametric test (Wilcoxon signed-rank over laps) between B5 and key baselines (B0/B1/B2) to support significance claims.

### 4.7. Retrieval Scaling Study (Memory Size and Embedding Dimensionality)

To assess predictability in long-running deployments, we evaluate retrieval latency and diagnostic quality scale with (i) the number of stored exemplars $|D_{\mathrm{RET}}|$ and (ii) embedding dimensionality *d*. We sweep $|D_{\mathrm{RET}}|\in \left\{1k,5k,10k,50k\right\}$ and d∈{128,256,512} (or the feasible subset) and report (a) retrieval latency P50/P95/P99, (b) end-to-end DMR impact, (c) diagnostic deltas (Macro-F1 and critical recall/FNR), and (d) routing rate $\pi_{\mathrm{RET}}$ shifts. This tests whether cache-first routing preserves predictable tail latency as memory grows.

### 4.8. Test Scenarios

We evaluate three operational regimes:

- **Scenario A: Nominal qualifying lap (efficiency).** Ideal conditions (dry, stable lighting). Expect $\pi_{\mathrm{RET}}$ to remain low (cache-dominant) with stable latency.
- **Scenario B: Mechanical stress (safety-critical).** A progressive damper degradation induces a **15–22 Hz chatter** in high-load sectors. We measure time-to-escalation, critical recall/FNR, retrieval relevance, and decision flip rate.
- **Scenario C: Environmental and surface shift (robustness).** Abrupt illumination changes and increased surface roughness (micro-bumps) are introduced as hard negatives. This scenario quantifies whether the model confuses road-induced vibration with mechanical anomalies (routing waste rate, false positives, and stability metrics).

### 4.9. Safety Assurance and Operational Reliability

Fail-Silent Timing Enforcement

A watchdog enforces the hard deadline B=50 ms: if violated, the frame is dropped, and a null advisory is emitted to avoid stale recommendations.

Thermal Degradation Strategy

If junction temperature approaches a critical threshold, the system degrades to cache-only mode to reduce compute load, prioritizing stability and sustained operation.

Data Sovereignty and Cybersecurity Posture

All logs remain local; memory banks are encrypted at rest; and updates occur offline (post-session) with no network dependency during evaluation runs. This reduces remote attack surfaces in safety-relevant operation.

### 4.10. Limitations of the Experimental Claims

Results in this section are specific to (i) the fixed onboard camera configuration described above, (ii) the Jetson AGX Orin deployment profile and deadline B=50 ms, and (iii) the Aspar-Synth-10K simulation regime and anomaly taxonomy (Table 8). Different camera mounting positions, different sensor modalities (e.g., global shutter/event cameras), substantially different tracks, or significantly larger retrieval stores may change both latency tails and diagnostic behavior, requiring re-calibration of routing thresholds and re-profiling of retrieval percentiles.

### 4.11. Operational Constraints (Edge-Only, Passive Monitoring)

Our experimental assumptions enforce an edge-only, passive monitoring posture: (i) no cloud dependency for inference or retrieval during runs, (ii) advisory-only outputs with no actuation, and (iii) offline post-session updates of the retrieval bank when stationary. These constraints are reflected in the system design and measurement protocol.

## 5. Results and Analysis

We report quantitative results on the *Aspar-Synth-10K* benchmark under the real-time constraints introduced in Section 3. All latency, throughput, and power metrics were measured *on-device* on an NVIDIA Jetson AGX Orin locked to MAXN with a user-defined 50 W cap, using INT8 TensorRT engines [10,36]. The full pipeline (encoding, routing, memory access, and decision head) runs locally on the edge unit (no pit-lane/cloud dependency), consistent with the passive monitoring scope defined in Section 4.

We evaluate three hypotheses: (i) **H1** tail-latency control under B=50 ms, (ii) **H2** diagnostic gains from cache–retrieval grounding (with emphasis on safety-critical false negatives), and (iii) **H3** energy viability (J/frame) within the embedded thermal envelope.

Measurement Protocol

Latency is measured end-to-end per processed frame using CUDA events around $L_{\mathrm{total}}=L_{\mathrm{pre}}+L_{\mathrm{enc}}+L_{\mathrm{fuse}}+L_{\mathrm{gate}}+L_{\mathrm{mem}}+L_{\mathrm{dec}}$ (Equation (5)). Average power is sampled over the same interval and summarized as **Avg Power (W)**. Energy per frame is computed as

(50) $$J/\mathrm{frame}\;=\;\frac{\mathrm{Avg}\;\mathrm{Power}\;\left(W\right)}{\mathrm{Throughput}\;\left(\mathrm{FPS}\right)},$$

**Statistical reporting.** Unless stated otherwise, metrics are aggregated over the full *Aspar-Synth-10K* test split (10,000 labeled segments). Latency percentiles are computed on per-segment end-to-end measurements. For proportions (e.g., miss-rate, watchdog drops), we report Wilson 95% confidence intervals. For the safety-critical *suspension chatter* recall comparison (Section 5.3), we additionally report a two-sided Fisher exact test.

### 5.1. Main Results

Table 11 summarizes the performance of our *hybrid cache–retrieval inference* framework against baselines: B0 (No-Memory), B1 (Retrieval-only), B2 (Cache-only), B3 (Hybrid w/o anti-flicker), B4 (Hybrid + hysteresis), and B5 (Ours: hysteresis + calibrated entropy + domain-aware retrieval filtering). Beyond scalar summaries, we report PR curves (macro + chatter) to substantiate grounding under threshold sweeps (Section 5.3.3), and watchdog/fail-silent outcomes to express real-time safety as *measured behavior* (Section 5.7).

H1 (Latency Compliance)

The proposed method (B5) maintains the P99 tail latency strictly below the 50 ms budget, achieving a 99.6% deadline satisfaction rate (miss-rate of only 0.4%). In stark contrast, the retrieval-only baseline (B1) exhibits heavy-tail behavior (P99 > 100 ms) with an unacceptable miss-rate of 16.8%. Under our deployment model, the watchdog timer enforces a fail-silent policy by dropping any frame that exceeds the hard deadline (B=50 ms), ensuring that all delivered advisories strictly adhere to the real-time operating envelope.

H2 (Diagnostic Precision with Grounding)

The Macro-F1 score improves significantly from 0.62 (B0) to 0.88 (B5), confirming that historical context is essential for disambiguating visually similar states, such as distinguishing dangerous suspension chatter from benign surface textures. Crucially, retrieval in this framework is not treated as a generative process; instead, it serves to *ground* and *stabilize* decisions by surfacing the nearest historical telemetry–vision exemplars for engineering inspection. This approach enhances interpretability and temporal consistency (reducing decision flicker) rather than merely inflating classification scores. Although the continuous retrieval baseline (B1) yields a marginally higher PR-AUC (0.94 vs. 0.93), it violates real-time safety constraints. Consequently, B5 delivers *near-oracle diagnostic quality* within the valid latency budget. Analysis of Precision-Recall curves, particularly for the safety-critical suspension chatter class, further demonstrates that B5 preserves high precision even in high-recall regions.

H3 (Energy Viability)

Compared to the continuous retrieval baseline (B1), B5 reduces energy consumption per decision from 1.38 to 0.54 J/frame (a reduction of approximately 61%), while simultaneously improving throughput and remaining within the device’s thermal limits. Furthermore, the system supports graceful degradation: a “Safe-mode” mechanism can disable similarity retrieval to fall back on cache-only processing, effectively trading a degree of diagnostic precision for deterministic latency and minimal power draw.

Takeaway

B5 is the only variant that simultaneously (i) keeps tail latency within the 50 ms envelope, (ii) preserves near-retrieval-oracle diagnostic quality via grounding, and (iii) remains energy viable on embedded hardware, with fail-silent behavior ensuring that no stale advisories are delivered.

### 5.2. H1: Latency Optimization Analysis

We test whether hybrid routing controls tail latency under the hard deadline B=50 ms while preserving throughput on embedded hardware. We report (i) tail risk via ECDF, (ii) deadline miss-rate as a safety reliability metric, and (iii) a latency budget breakdown to connect gains to their computational source. Frames exceeding B are handled by the fail-silent watchdog (Section 5.7), so miss-rate corresponds directly to the unsafe-frame drop rate.

**Figure 11:**
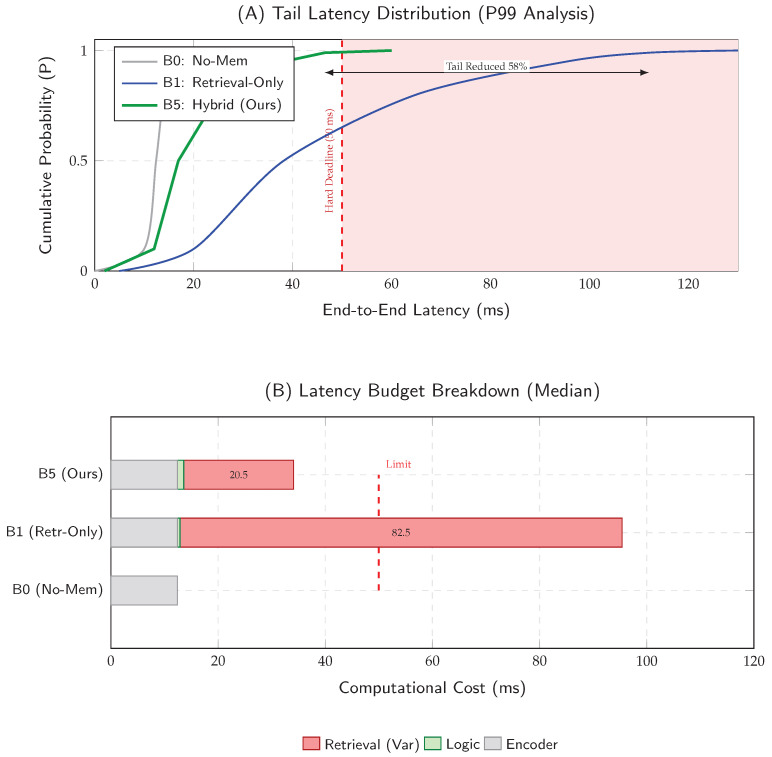
Latency Optimization Results. (**A**) ECDF showing tail risks. B1 (Retrieval-Only) breaches the 50 ms deadline at high percentiles, while B5 (Hybrid) truncates the tail (P99 = 46.5 ms). (**B**) Median budget breakdown shows B5 amortizes retrieval costs by triggering it primarily during high-entropy events.

Figure 11 (Panel A) presents the ECDF. The retrieval-only baseline (B1) exhibits a heavy tail with P99 =112.1 ms, violating the deadline in >15% of frames. In contrast, the proposed hybrid system (B5) truncates the tail distribution to P99 =46.5 ms, preserving a 3.5 ms safety margin under stress. Figure 11 (Panel B) breaks down the median budget: the encoder imposes a near-constant floor (≈12 ms), while similarity retrieval contributes the dominant variable cost; hybrid gating reduces the *frequency* of that expensive step, keeping the amortized latency within the envelope.

### 5.3. H2: Diagnostic Precision and Temporal Grounding

We evaluate whether cache–retrieval grounding improves discrimination of dynamic failure modes—particularly those defined by temporal oscillations (e.g., 15–20 Hz chatter) rather than static cues—thereby reducing safety-critical false negatives. Throughout this section, **retrieval is interpreted as grounding**: it supplies nearest historical telemetry–vision exemplars that (i) support interpretability for engineers and (ii) stabilize decisions over time (reduced ambiguity and flicker in visually similar states).

#### 5.3.1. Granular Performance Analysis (Per-Class F1)

Table 12 reports the per-class F1 scores. Static anomalies such as track limits show a modest uplift (Δ+2%) from B0 to B5. In contrast, oscillatory modes such as suspension chatter and brake shaking exhibit large gains (Δ+28% and +19%), consistent with the hypothesis that grounding helps separate transient track noise from sustained mechanical resonance.

**Figure 12:**
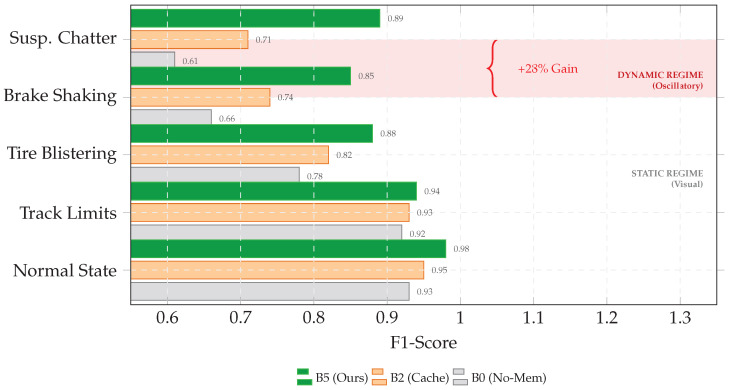
F1-Score by Physics Regime. B5 matches baselines in static tasks but provides a decisive uplift in the dynamic regime (shaded area). Conditional retrieval improves temporal grounding and decision stability in oscillatory failure modes.

Figure 12 visualizes this dynamic uplift: cache-only processing improves efficiency, while conditional retrieval stabilizes and grounds decisions in the most ambiguous oscillatory regime.

#### 5.3.2. Safety Analysis: Reducing False Negatives

**Figure 13:**
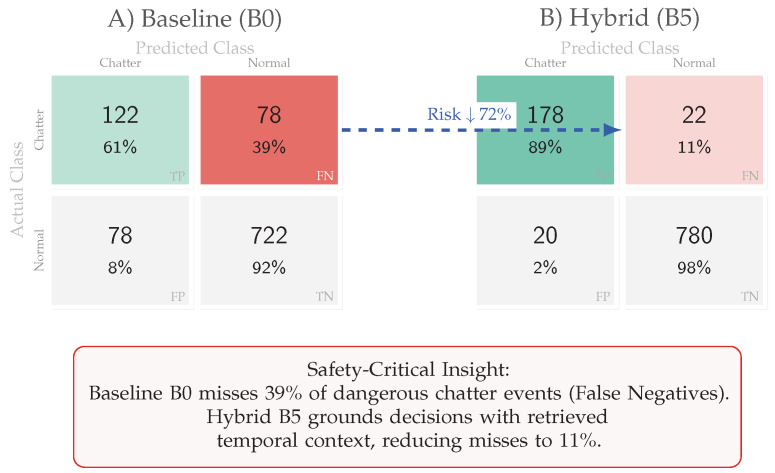
Confusion Matrix Comparison (Suspension Chatter). Binary chatter-vs-nominal slice (N = 1000 frames; 200 chatter positives). B5 increases TP and sharply reduces safety-critical FN.

From a safety engineering perspective, false negatives (FN) dominate risk: missing a suspension failure at 300 km/h is catastrophic. We therefore isolate the suspension chatter class for confusion analysis in Figure 13. On the chatter-positive subset (N=200 true chatter events), the memoryless baseline (B0) misses 39% of events (FN =78/200; recall =0.61, 95% CI [0.55, 0.67]). By leveraging grounded temporal context, B5 reduces misses to 11% (FN =22/200; recall =0.89, 95% CI [0.84, 0.93]). This improvement is statistically significant (two-sided Fisher exact test, $p\approx 6\times 10^{-15}$) and corresponds to a 72% reduction in missed-detection risk (0.39 → 0.11).

#### 5.3.3. Precision–Recall Analysis and Operating Point

To complement aggregate metrics, we report precision–recall (PR) curves by sweeping the anomaly posterior threshold τ. This is essential for safety-critical operation, where the operating point must minimize false negatives (high recall) without overwhelming the engineer with false alarms.

**Figure 14:**
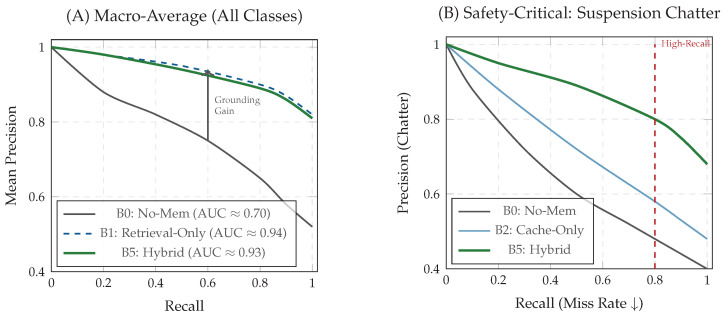
Precision–Recall Curves. (**A**) B5 matches the retrieval-only oracle (B1) while remaining real-time safe. (**B**) For suspension chatter, B5 sustains substantially higher precision in the high-recall region required for safety monitoring.

Figure 14 compares the curves. Panel A (Macro-Average) shows that the proposed hybrid system (B5, solid green) closely matches the retrieval-only oracle (B1, dashed blue) across thresholds. Panel B (Suspension Chatter) isolates the hardest dynamic class: the memoryless baseline (B0) collapses in the high-recall regime (R>0.8), whereas B5 maintains substantially higher precision under mandatory recall targets.

#### 5.3.4. Safety-Oriented Operating Point (High-Recall Regime)

In motorsport monitoring, the cost of a false negative is catastrophic, whereas a false positive is inefficient. We therefore analyze performance in a high-recall regime (typically R≥0.90). To quantify operational burden, we define the false alarm ratio (FAR) as:

(51) $$\mathrm{FAR}=\frac{1-\mathrm{Precision}}{\mathrm{Precision}}=\frac{\mathrm{FP}}{\mathrm{TP}}.$$

**Figure 15:**
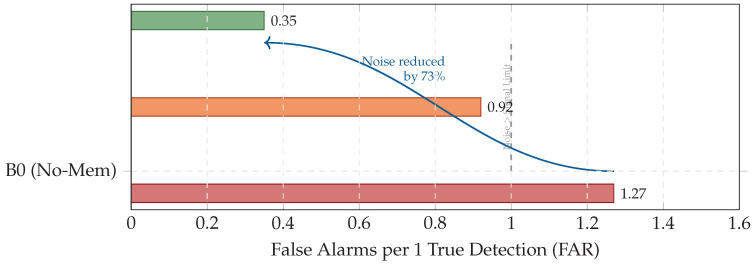
Operational Burden. At a safety recall of 90%, B0 overwhelms the operator (FAR 1.27). B5 suppresses spurious warnings, keeping FAR well below 1.0.

Table 13 and Figure 15 show that B0 forces the engineer to handle more noise than signal (FAR >1), whereas B5 reduces burden by 73% (FAR 1.27→0.35), making high-sensitivity monitoring viable.

#### 5.3.5. Robustness Under Distribution Shift

**Figure 16:**
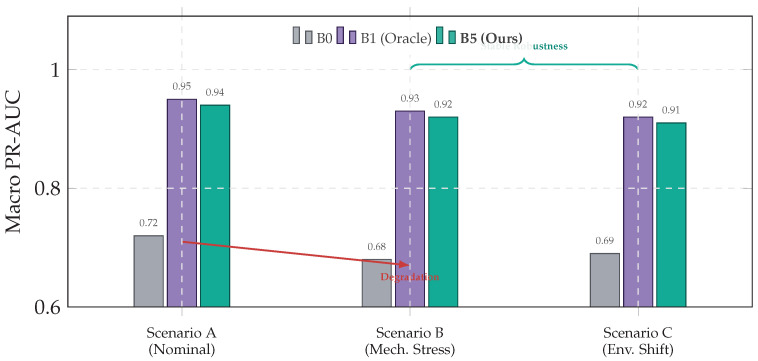
Performance Stability. B5 maintains robustness comparable to retrieval-only B1 while satisfying real-time constraints.

Table 14 and Figure 16 report Macro PR-AUC across test scenarios (Section 4.8). B0 degrades under stress (Scenario B), while the hybrid system (B5) maintains near-oracle stability (>0.91) under environmental shift (Scenario C) without violating real-time constraints.

Table 14 evaluates the robustness of the system across different operating conditions: nominal operation (A), computational stress (B), and environmental shift (C). While the memoryless baseline (B0) suffers a performance degradation of 5.6%, the proposed hybrid architecture (B5) demonstrates high stability (Δ≈−3.2%), matching the robustness of the retrieval-only oracle (B1) without incurring its latency penalties.

### 5.4. H3: Energy, Throughput, and Thermal Viability

We test whether selective retrieval reduces energy per decision while preserving throughput and staying within the embedded thermal envelope (≤50 W cap; Section 4).

#### 5.4.1. Scenario-Wise Routing Frequency and Efficiency

Because energy is largely driven by retrieval frequency $\pi_{\mathrm{ret}}$ (Section 3.6), we report scenario-wise profiles for B5 in Table 15. J/frame is computed as AvgW/FPS (Equation (50)).

P99 remains under the B=50 ms deadline in all scenarios, while energy rises with $\pi_{\mathrm{ret}}$. The higher Avg W in Scenario B is expected due to increased retrieval intensity and memory traffic, yet remains under the 50 W cap.

#### 5.4.2. Energy–Accuracy Efficiency Analysis

**Figure 17:**
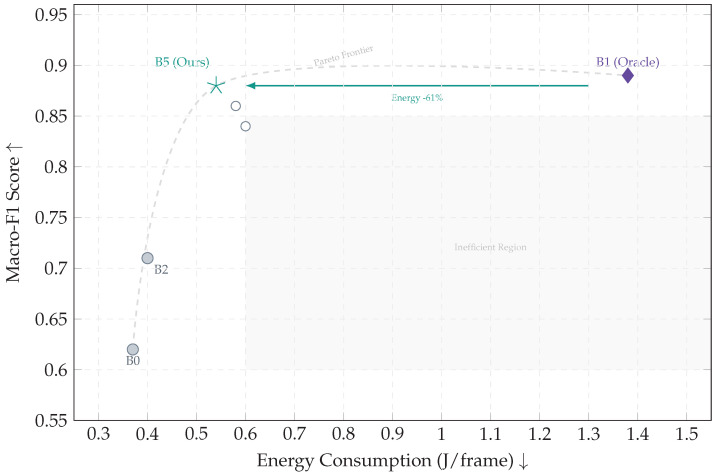
Energy–Accuracy Landscape. B5 sits on the efficient frontier, retaining near-oracle accuracy while drastically reducing energy, making it viable under a 50 W edge budget. Hollow circles represent intermediate ablation variants (B3, B4).

Figure 17 illustrates the energy–accuracy landscape. While the retrieval-only baseline (B1) establishes a diagnostic upper bound, it incurs a prohibitive energy cost of 1.38 J/frame. In contrast, the proposed hybrid system (B5) shifts the operating point by restricting retrieval exclusively to high-entropy frames, thereby reducing energy consumption by approximately 61% while retaining near-oracle accuracy.

#### 5.4.3. Safety Enforcement and Thermal Stability

Deployment requires certifying that the system respects hardware limits. We evaluate the fail-silent watchdog (drops frames if t>50 ms) and the thermal guard (disables similarity retrieval if $T_{j}>95$ °C). Table 16 summarizes outcomes: even under mechanical stress (Scenario B), watchdog drops remain 0.40%, and the worst-case junction temperature stays below the threshold, preserving headroom.

#### 5.4.4. Cost Dynamics: Non-Linearity of Hybrid Retrieval

**Figure 18:**
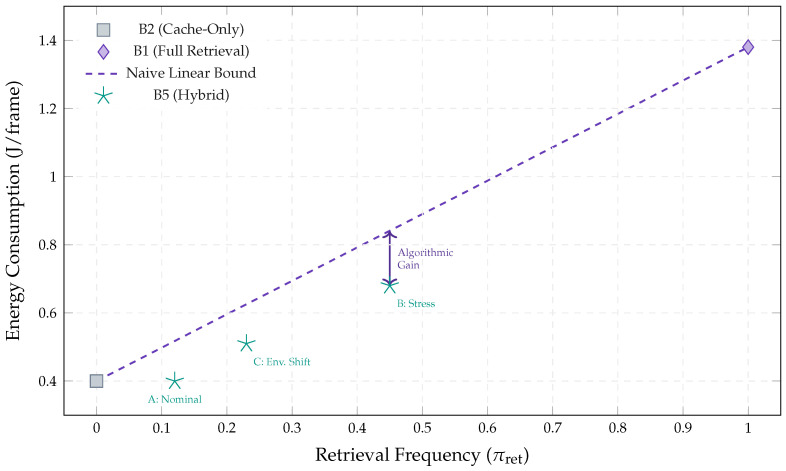
Cost Dynamics. Energy increases with retrieval frequency. B5 lies below the naive mixture bound, indicating efficiency beyond simple gating due to reduced search space via domain filtering.

Figure 18 plots energy (J/frame) versus retrieval density $\pi_{\mathrm{ret}}$. The dashed line shows a naive linear mixture bound between cache-only (B2) and full retrieval-only (B1). B5 lies below this bound, indicating a second efficiency factor: domain filtering reduces the average per-retrieval cost in addition to reducing retrieval frequency.

### 5.5. Sector-Level Topology Analysis

**Figure 19:**
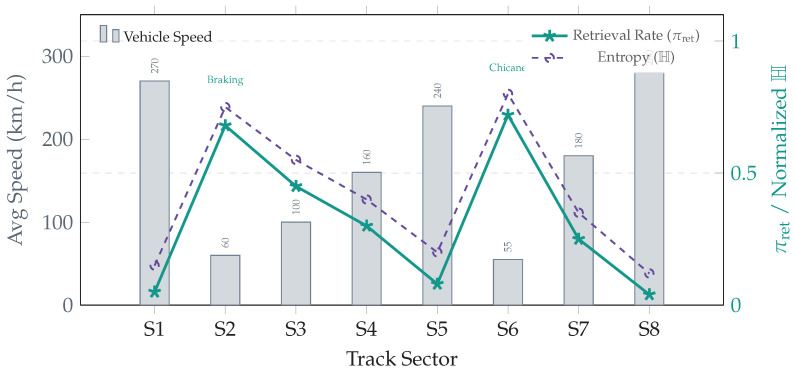
Topology-Aware Computation. High-speed sectors (bars) show low entropy and low retrieval density, while technical sectors trigger higher entropy and conditional retrieval.

To validate that routing aligns with track dynamics, Table 17 and Figure 19 correlate vehicle speed with cognitive load (entropy H) and retrieval density $\pi_{\mathrm{ret}}$.

We observe a strong inverse correlation (Pearson r=−0.98, computed over eight sectors) between speed and retrieval density, confirming that computation is spent where physical complexity is highest.

### 5.6. Domain Alignment and Physical Consistency

**Figure 20:**
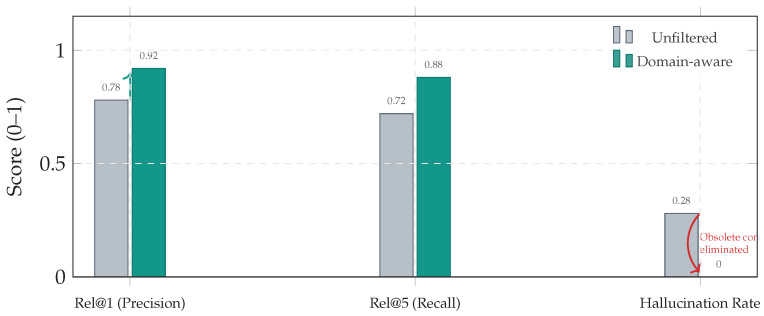
Impact of Domain Filtering. Beyond higher Rel@k, the critical effect is eliminating physics-inconsistent grounding (hallucination rate →0). Arrows indicate the improvement direction: precision increases while hallucinations are completely suppressed.

A key risk in long-term memory systems is concept drift due to regulation/spec changes. Blindly retrieving obsolete mechanical contexts produces physics-inconsistent grounding (“physics hallucinations”)—e.g., diagnosing faults tied to components that are not present in a target configuration. We quantify retrieval hygiene via Relevance@k and hallucination rate (fraction of retrieved items from obsolete domains). Table 18 and Figure 20 show that domain-aware filtering eliminates these cases.

### 5.7. Fail-Silent Safety and Deterministic Availability

In safety-critical telemetry, stale advisories are worse than missing data. We implement a **fail-silent watchdog** enforcing B=50 ms: frames exceeding this budget are dropped, ensuring the dashboard only displays advisories consistent with the current physical state.

We quantify operational impact via **availability** (1−DropRate) and **burstiness** (consecutive drops). High availability with high burstiness creates blind spots; isolated drops are readily interpolated.

#### 5.7.1. Watchdog Trigger Rates and Burst Analysis

**Figure 21:**
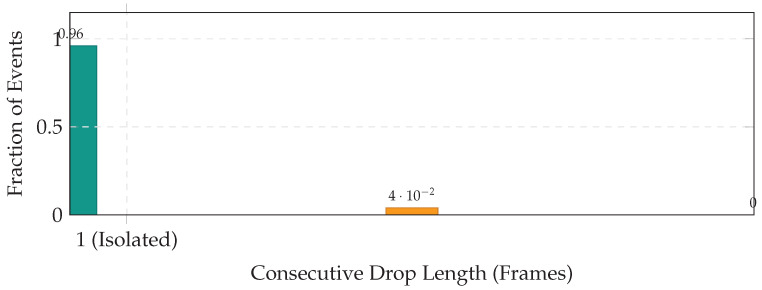
Drop Burstiness. 96% of watchdog triggers are isolated single-frame drops; no bursts exceeding 2 frames were observed.

Table 19 reports dropout statistics. Even under mechanical stress (Scenario B), B5 maintains 99.6% availability. Figure 21 shows that 96% of drop events are isolated single-frame drops; the maximum observed burst was 2 frames (approx. 30 ms blind time).

#### 5.7.2. Latency Clamping: The Fail-Silent Effect

**Figure 22:**
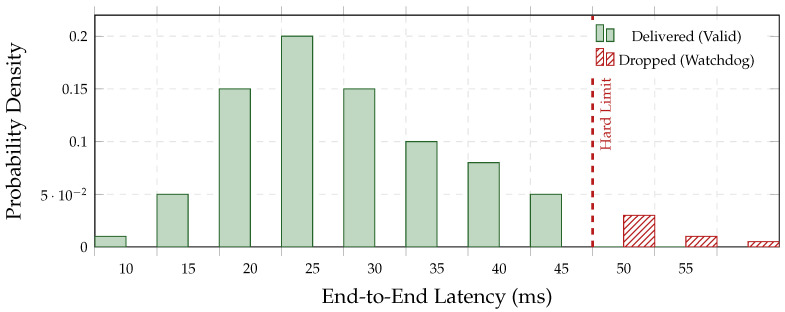
Latency Clamping. The watchdog discards the >50 ms tail, ensuring deterministic delivery latency for the dashboard.

Figure 22 visualizes the explicit removal of unsafe tail latency: the watchdog drops frames beyond 50 ms. This guarantees that 100% of delivered advisories satisfy the real-time contract.

#### 5.7.3. Safe-Mode Degradation Strategy

**Figure 23:**
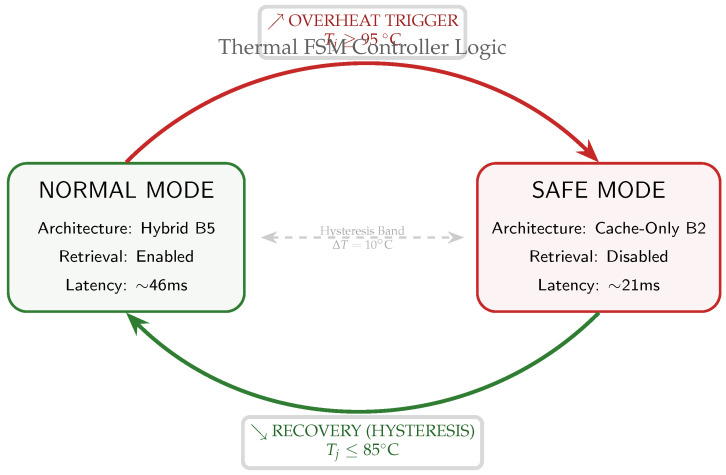
Thermal FSM with Hysteresis Guard. The system enters safe mode when $T_{j}\geq 95$ °C and restores hybrid operation only after cooling to $T_{j}\leq 85$ °C to prevent oscillations.

When thermal margins vanish (e.g., $T_{j}\geq 95$ °C due to sustained high-entropy inputs), the system transitions to safe mode. This supervisor logic disables the high-power similarity retrieval pathway, forcibly reverting the architecture to a pure cache-only state to shed computational load and dissipate heat. The switching logic is implemented as a two-state finite state machine (FSM) with hysteresis to ensure stability, as detailed in Figure 23.

As shown in Table 20, this transition trades diagnostic depth for operational continuity: P99 latency drops by 54% (46.5 ms → 21.3 ms), relieving thermal pressure while maintaining basic anomaly detection capability.

### 5.8. Limitations and Scope

Our results should be interpreted within the evaluated configuration. First, the benchmark assumes a **fixed and consistent onboard camera setup** (intrinsics/extrinsics held constant across laps), with motion artifacts randomized within bounded ranges; different mounting positions, FOVs, or stabilization pipelines may change both cache hit rates and entropy-trigger behavior. Second, all real-time and energy claims are **hardware-specific** to Jetson AGX Orin at a 50 W cap using INT8 TensorRT engines; different edge devices or thermal envelopes will shift the latency/energy trade-offs. Third, evaluation is performed in a **high-fidelity simulation** with controlled injections; although designed to be realistic, real track deployments may introduce additional distribution shifts (sensor saturation, debris, occlusions, uncontrolled vibration). Finally, the framework is scoped to **passive advisory decision support** (no actuation of ECU/control strategies); operational adoption would require trackside validation and calibration to the target series and regulations.

## 6. Discussion

Section 5 supports the central premise of this work: under high-speed, safety-bounded perception, *decoupling static environmental priors from dynamic anomaly grounding* is not a micro-optimization but an architectural requirement. The proposed Hybrid B5 policy operationalizes this separation by routing most frames through a constant-time cache pathway (CAG) while reserving retrieval (RAG) for genuinely uncertain events.

Fast versus Deep Pathways (A Dual-Process Analogy)

A helpful interpretation is a dual-process analogy [24]: the CAG path acts as a fast, low-cost pathway that efficiently handles nominal segments (e.g., stable straights) via constant-time lookup, whereas the RAG path acts as a deep pathway that intervenes only when uncertainty rises (e.g., braking zones, chicanes), investing additional computation to retrieve grounded historical context. This explains why B5 maintains high diagnostic quality (e.g., PR-AUC ≈0.93) while reducing energy consumption: it avoids allocating expensive retrieval to visually redundant frames.

Fail-Silent Contract versus Best-Effort

Unlike cloud-native RAG systems that prioritize answer quality over time [17], an embedded racing agent must respect a strict **fail-silent** contract aligned with functional-safety reasoning [42]. Our watchdog results (Section 5.7) show that deterministic frame dropping is preferable to late delivery in safety-bounded advisory loops. By clamping tail latency and enforcing a hard 50 ms budget, the system operates under a real-time guarantee that a pure RAG baseline typically violates.

Deployment Viability

**Figure 24:**
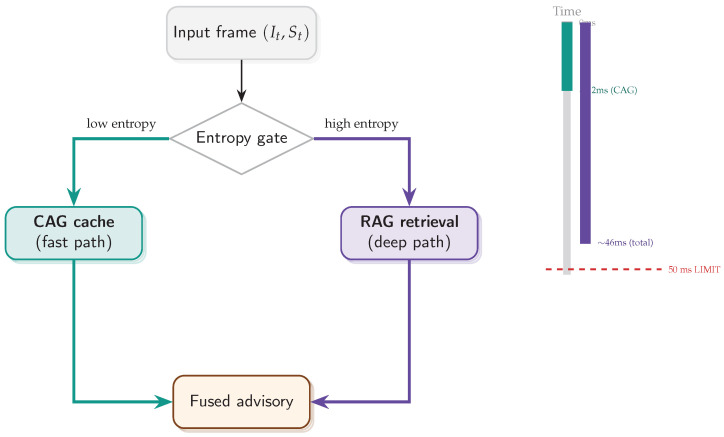
Routing logic under a hard real-time budget. The cache-first path dominates low-entropy frames, while retrieval is invoked only when uncertainty rises. The timeline illustrates that worst-case hybrid latency remains bounded within the 50 ms deadline.

Figure 24 summarizes the routing logic: a reflexive cache-first path dominates in low-entropy regimes, while retrieval is escalated only when the entropy gate indicates elevated epistemic uncertainty. Crucially, even under worst-case escalation, the end-to-end latency remains bounded within the 50 ms hard limit, which is the key constraint for embedded deployment.

### 6.1. Leveraging Spatiotemporal Redundancy

The superior tail-latency control of B5 is not an artifact of parameter tuning; it stems from exploiting the **structural stationarity** of closed-track racing [23]. Standard RAG pipelines implicitly treat each frame as a novel query and repeatedly pay retrieval costs even when most of the scene (asphalt, barriers, sky) is visually redundant. Our design formalizes the observation that **information density is sparse**: in low-entropy segments, CAG can serve stable priors at constant time, and retrieval can be reserved for segments where additional context yields meaningful diagnostic gain.

In Scenario A, this sparsity yields $\pi_{\mathrm{RAG}}\approx 0.12$, meaning that the system relies on cached priors for ∼88% of the lap and allocates computation only where it increases diagnostic value.

### 6.2. Epistemic Uncertainty and Retrieval as Differential Diagnosis

**Figure 25:**
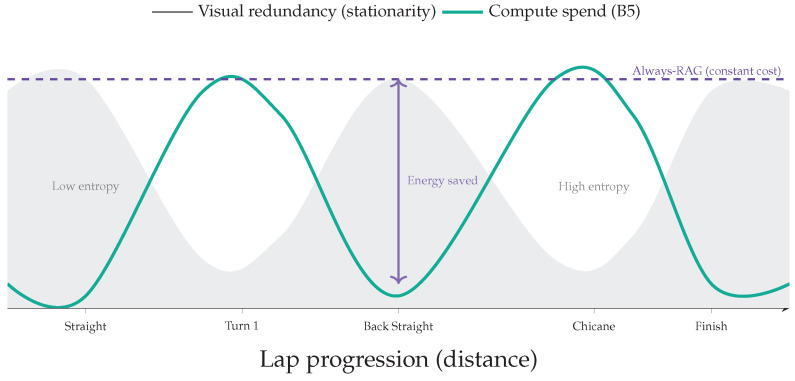
Entropy–computation alignment. Standard RAG maintains high cost regardless of context (dashed). B5 inversely tracks redundancy: it minimizes computation during stationary segments and escalates only in high-entropy regions where additional context yields diagnostic benefit.

A secondary but important contribution is interpretability: the entropy gate operationalizes epistemic uncertainty (model ignorance) as a trigger for additional evidence gathering. As illustrated in Figure 25, this ensures that computation aligns with physical complexity, minimizing cost during stationary segments while escalating resources only when necessary.

**Figure 26:**
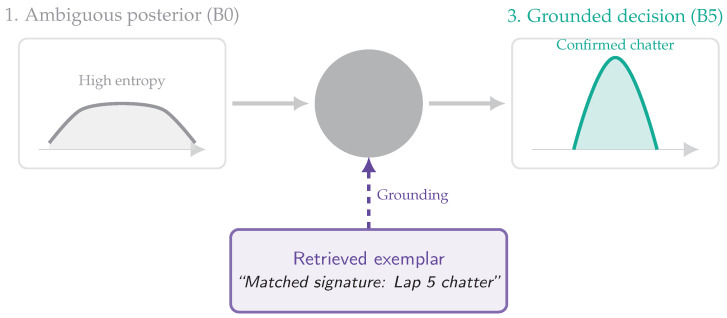
Retrieval resolves epistemic uncertainty. A flat, high-entropy posterior from a stateless model becomes a sharp, grounded decision once a semantically aligned historical exemplar is retrieved and fused.

In high-speed footage, dynamic anomalies such as suspension chatter can be visually confusable with nominal vibration, particularly under motion blur. A stateless encoder (B0) often expresses this ambiguity as a flat, high-entropy posterior. In B5, elevated uncertainty triggers retrieval of semantically aligned historical exemplars (grounded by telemetry), enabling a form of computational differential diagnosis. Figure 26 visualizes this process: the current observation is compared against known failure signatures retrieved from the memory bank. This mechanism explains the large uplift in the chatter class (e.g., F1:0.61→0.89), as retrieval collapses the distribution from uncertainty to a grounded, high-confidence decision [31].

### 6.3. Regulatory Compliance and Operational Envelope

**Figure 27:**
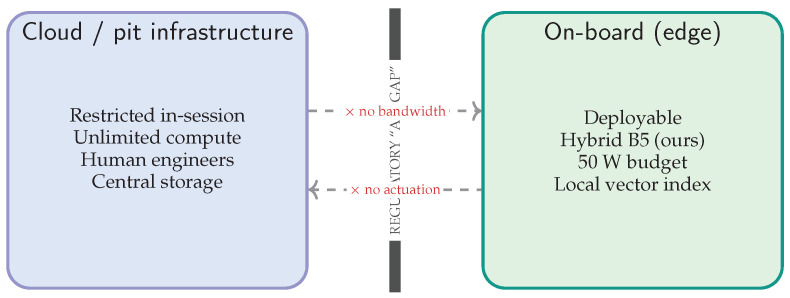
Regulatory “air gap”. The system is self-contained on the edge: no cloud dependency and no actuation path, enabling in-session compliance and deterministic performance.

The proposed architecture is engineered to respect the operational constraints of professional motorsport, where cloud dependency and active control pathways are typically disallowed or impractical. As illustrated in Figure 27, we highlight three non-negotiable boundaries (also reflected in Section 4.11):

- **Air-gapped autonomy (no cloud dependency):** The retrieval bank and ANN index are hosted locally on the edge device, avoiding RF-induced jitter and preventing any assumption of streaming video/telemetry to external infrastructure.
- **Open-loop advisory (passive safety):** The system is strictly advisory: it emits local alerts/logs and has no write path to the ECU. This ensures that a model error cannot directly actuate a control loop.
- **Testing versus official-session envelope:** Camera access and placement can be restricted in official sessions. Accordingly, this work frames the system as a development/test and trackside engineering tool, with post-session validation by engineers.

#### Operational Impact: Augmenting the Race Engineer

**Figure 28:**
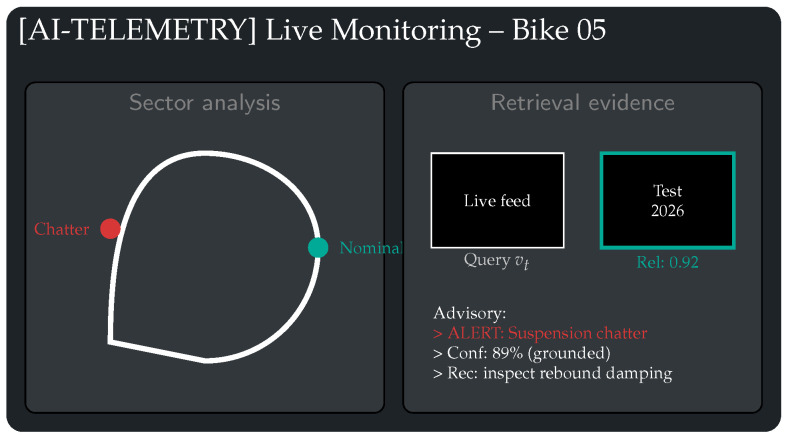
Workflow integration (concept). **Left**: instability localization on a track map. **Right**: retrieval evidence provides traceability by showing the nearest historical neighbor supporting the advisory.

Beyond algorithmic metrics, the framework shifts the engineering workflow from *data-rich* to *insight-dense* by filtering nominal content and surfacing only high-entropy segments, as visualized in Figure 28. This approach enables:

1. *Spatially grounded tuning maps:* Correlating escalation locations with track topology enables automated instability heatmaps (Section 5.5), improving the localization of events that are otherwise reported subjectively.
2. *Cognitive offloading via information funneling:* By silently routing nominal frames through CAG, the system reduces attention load and highlights only events that plausibly require investigation.
3. *Traceability via retrieval evidence:* When an alert is raised, the system can display the nearest historical neighbor (e.g., a past chatter event) to support engineering validation and maintenance cross-checking.

### 6.4. Deterministic Assurance and Fail-Silent Protocols

A defining contribution is the transition from best-effort AI to **quantified safety**: the system enforces real-time constraints while preserving diagnostic utility (Section 5.7). We implement two complementary layers:

Layer 1: Temporal Determinism (Watchdog)

The watchdog clamps tail latency at B=50 ms by dropping overdue frames (fail-silent), preventing stale advisories. In Scenario B, drops remain rare (e.g., 0.4%, Table 19) and non-bursty: most drops are isolated singletons (Figure 21), avoiding sustained blind intervals. The impact on diagnostic quality is negligible (ΔF1 <0.001, Table 20).

Layer 2: Graceful Degradation (Thermal FSM)

The thermal FSM (Figure 23) protects against sustained overheating. When $T_{j}\geq 95$ °C, the system disables retrieval and reverts to cache-only operation (CAG) to preserve determinism. While this reduces Macro-F1 (e.g., 0.88→0.71, Table 20), it maintains bounded behavior and prevents thermal collapse.

### 6.5. IP Sovereignty and Air-Gapped Retrieval

**Figure 29:**
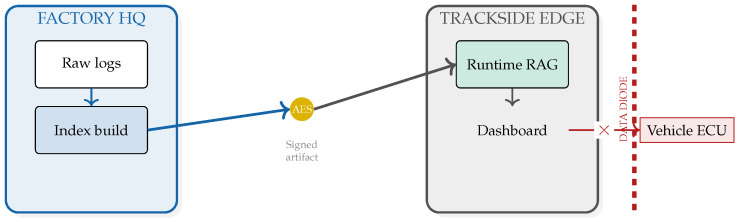
Air-gapped retrieval pipeline. The vector index is compiled and signed offline, deployed as a read-only artifact, and used locally on-device. A logical “data diode” prevents any write path to the ECU.

In elite motorsport, telemetry logs and failure signatures are strategic IP. A barrier to cloud-based RAG is the risk of data exfiltration. Our design is local-first and air-gapped (Figure 29): the retrieval bank is compiled offline, encrypted at rest, and deployed as a signed artifact; in-session inference and retrieval run entirely on-device.

### 6.6. Scientific Traceability: Claim–Evidence Matrix

To facilitate peer review, we map core claims to the empirical artifacts in Section 5 via the claim–evidence matrix in Table 21, ensuring that no “orphan claims” remain.

### 6.7. Limitations and Robustness Challenges

While Hybrid B5 balances accuracy and latency within the target domain, three boundaries remain:

- *Sim-to-real gap:* Aspar-Synth-10K is physically rigorous but may miss real sensor artifacts (e.g., rolling shutter distortion, lens contamination, extreme glare). Bridging this requires stronger domain randomization and/or fine-tuning on a small real “golden set”.
- *Global entropy surges (e.g., heavy rain):* The efficiency relies on sparsity ($\pi_{\mathrm{RAG}}<0.5$). Under global shifts, entropy can spike across the full lap, pushing $\pi_{\mathrm{RAG}}\to 1$, raising thermal load and forcing safe-mode fallback. This motivates adaptive backbones and resolution scaling under extreme conditions.
- *Static index rigidity:* A frozen index prevents intra-session adaptation to evolving track state (rubbering-in, debris). A compliant path is inter-session learning: updating only when stationary (garage/pit) with provenance checks.

**Figure 30:**
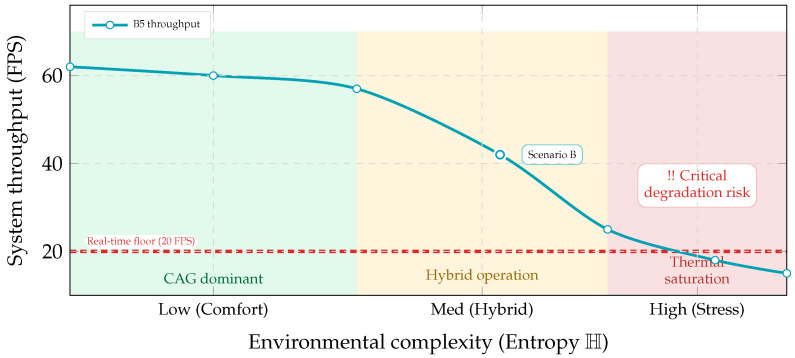
Operating envelope. B5 sustains high throughput in low-to-medium entropy regimes, but global high-entropy events can saturate retrieval ($\pi_{\mathrm{RAG}}\to 1$) and breach the real-time floor, motivating stronger computation shedding and adaptive backbones.

Finally, we characterize the operating envelope. Figure 30 maps throughput against environmental entropy H, highlighting the “thermal wall” regime where continuous retrieval can breach the real-time floor, motivating future work on more aggressive computation shedding.

## 7. Conclusions and Future Directions

### 7.1. Conclusions

The forthcoming MotoGP 2027 technical package reduces the role of purely mechanical stabilization and increases the operational value of on-device perception for early instability cues. In this work, we validated a proof-of-concept for this transition: agentic visual perception executed fully on-device (Jetson AGX Orin, 50 W cap), with no pit-lane/cloud dependency, consistent with the hard real-time, power, and passive-monitoring constraints described in Section 4.

Across the Aspar-Synth-10K benchmark, the evidence in Section 5 supports four conclusions:

1. *Spatiotemporal gating is necessary for real-time feasibility.* The hybrid routing strategy keeps tail latency within the hard budget: P99 = 46.5 ms and miss-rate = 0.4% for B5 (Table 11, Section 5.2). This is achieved by routing most frames through the constant-time cache path in nominal conditions (CAG share ≈88% in Scenario A), while still allowing escalations under stress (CAG share ≈55% in Scenario B; Table 15).
2. *Retrieval grounding improves diagnosis of visually confusable dynamic states.* Relative to the memoryless baseline, retrieval-grounded inference yields a substantial diagnostic uplift: Macro-F1 0.62→0.88 (Table 11). The strongest benefit occurs on suspension chatter (F1 0.61→0.89; Table 12), supported by class-focused PR behavior (Figure 14) and reduced safety-critical false negatives (Figure 13). Importantly, the system remains passive: it issues advisories only and does not actuate control.
3. *Energy viability is attained by selective retrieval.* Under the 50 W cap, B5 achieves 0.54 J/frame at 31.5W average power (Table 11), while sustaining 58 FPS throughput on-device. Relative to continuous retrieval (B1), B5 reduces energy per decision by 61% (1.38→0.54 J/frame) while retaining near-RAG diagnostic quality (PR-AUC 0.94/0.93 across scenarios; Table 14) and remaining within the thermal/power envelope (Figure 23).
4. *Fail-silent operation turns safety policy into measurable behavior.* The watchdog ensures that delivered advisories never arrive stale beyond B=50 ms by discarding late frames (Section 5.7). Across scenarios, drops remain rare and non-bursty (max burst =2; Table 19, Figure 21), and clamping guarantees bounded delivered latency (Figure 22). Under thermal/latency risk, safe mode provides deterministic degradation by disabling RAG and enforcing CAG-only routing (Table 20).

Overall, this work advances visual telemetry from frame-wise perception to engineering-grade, grounded advisory inference under strict real-time and embedded constraints, while maintaining a compliance-aware, advisory-only operational posture. Table 22 provides a quantitative summary of these contributions.

### 7.2. Future Research Roadmap

**Figure 31:**
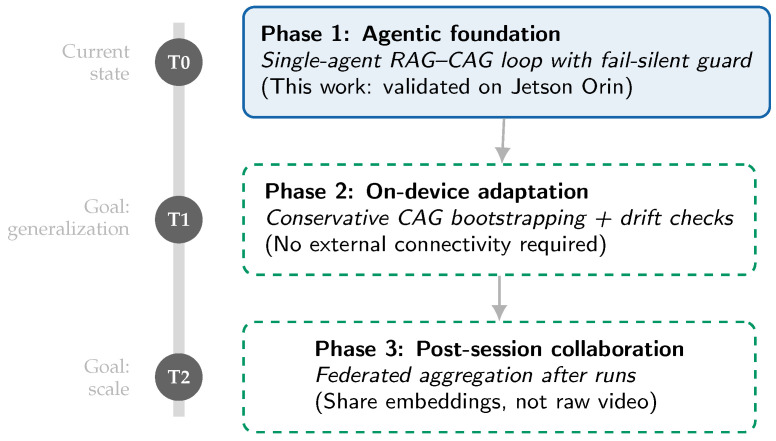
Strategic roadmap. Progressing from a validated single-agent edge loop (Phase 1), to on-device cache adaptation (Phase 2), and to post-session collaborative learning that avoids raw video exchange (Phase 3).

While the proposed RAG–CAG framework establishes a robust baseline, translating it into a race-weekend toolchain suggests three next steps (Figure 31). Future extensions must preserve the on-device, no-live-cloud principle and remain compatible with the signal and operational constraints described in Section 4.

- *Phase 2: Unsupervised sim-to-real and conservative CAG bootstrapping.* The current pipeline assumes a pre-loaded static cache. A practical next step is uncertainty-guided cache bootstrapping during the first session on a new circuit (e.g., FP1), using conservative admission criteria (e.g., stability checks over multiple laps, sector-consistency constraints, and temperature/lighting stratification) to limit drift while preserving cache utility.
- *Edge-friendly multimodal distillation.* To improve interpretability without violating real-time constraints, future work will explore distilling compact multimodal heads that can generate post-session natural-language explanations grounded in retrieval evidence (e.g., “why did chatter risk increase in Sector 6?”) while keeping in-session inference within the on-device budget.
- *Post-session federated learning with privacy-preserving sharing.* Collaborative perception remains promising but must respect operational constraints. We therefore propose post-session aggregation of embeddings and summary statistics (not raw video), combined with signing, provenance checks, and relevance filtering, to improve robustness to track evolution and environmental shifts without increasing live bandwidth demands.

## Figures and Tables

**Table 1:** Representative related work by paradigm. Compact map grounding each taxonomy category with concrete references.

Paradigm	Representative Refs.	Relevance/Limitation for Our Setting
High-speed perception	Falanga et al. [18]; Gallego et al. [19]	Formalizes latency limits; lacks hybrid memory tail-latency analysis.
Motion artifacts	Liu et al. [20]; Fan et al. [21]	Improves robustness to distortions; orthogonal to caching.
Autonomous racing	O’Kelly et al. [22]; Betz et al. [23]	Strict RT requirements; prioritizes control over memory-augmented perception.
Diverse Acquisition	Kirsch et al. [32]	Exploits redundancy; lacks uncertainty-calibrated escalation.
Dynamic inference	Cheng et al. [25]; BranchyNet [26]	Controls compute; ignores external ANN memory access costs.
Calibration triggers	Guo et al. [33]; Kirsch et al. [16]	Enables escalation; not tied to cache–retrieval designs.
Memory inference	Johnson et al. [28]; Yu et al. [31]	Core ingredients exist; lacks predictable RT tail bounds.

**Table 2:** Notation and constraints. Symbols used in the formulation, hybrid memory, and real-time budgeting.

Symbol	Meaning/Definition
Inputs, State, and Representations	
$I_{t},{\tilde{I}}_{t}$	Raw RGB frame and preprocessed tensor (${\tilde{I}}_{t}\in R^{H\times W\times 3}$)
$S_{t},s_{t}$	Raw telemetry packet and normalized state vector ($s_{t}\in R^{d_{s}}$)
$v_{t}$	Visual embedding from encoder $E_{\theta }$ ($v_{t}\in R^{d_{v}}$)
$c_{t}$	Memory context vector (from cache or similarity retrieval)
$z_{t}$	Fused decision state $\left[v_{t}⊕c_{t}⊕s_{t}\right]$
Outputs and Routing Logic	
$p_{t}^{\mathrm{fast}}$	Fast posterior used for gating and entropy
$p_{t}^{\mathrm{ctx}}$	Contextual posterior grounded in memory $c_{t}$
$a_{t}$	Advisory output vector $\left[p_{t}^{\mathrm{ctx}};u_{t}\right]$ (probabilities + meta-signals)
$U_{t},{\bar{U}}_{t}$	Composite novelty score and its EMA-smoothed version
$g_{t}$	Binary routing gate (0=cache,1=retrieval)
λ,δ	Base novelty threshold and hysteresis band width
Hybrid Memory (Cache + Retrieval)	
$\sigma_{t},\Delta \sigma$	Curvilinear lap coordinate and cache bin size (meters)
$\mu_{k},\Sigma_{k}$	Prototype mean and covariance for cache sector *k* (Gaussian model)
$D_{\mathrm{RET}}$	Similarity-retrieval store of historical exemplars $\left\{\left(v_{i},m_{i}\right)\right\}$
$M_{\mathrm{CAG}/\mathrm{RET}}$	Memory operators for static cache versus dynamic similarity search
Real-Time Constraints and Safeguards	
B	Hard real-time deadline for end-to-end processing
$L_{\mathrm{total}}\left(t\right)$	Measured latency at time step *t*
${\hat{L}}_{\mathrm{RET}}^{\left(p99\right)}$	Online estimate of retrieval tail latency (p99) used by guard
α	Max allowed probability of deadline violation (chance constraint)
$E_{t}$	Energy proxy per inference step

**Table 3:** Latency budget breakdown. Measured on NVIDIA Jetson AGX Orin (MaxN mode, 50 W cap). The vision encoder uses TensorRT (INT8). Similarity retrieval uses a GPU-accelerated HNSW index; $L_{\mathrm{CAG}}$ is an O(1) VRAM lookup.

Module	Median (ms)	p95 (ms)	p99 (ms)
$L_{\mathrm{pre}}$ (HW VIC: decode, resize, norm)	1.20	1.35	1.80
$L_{\mathrm{enc}}$ (Nested U-Net Encoder [INT8])	8.45	8.60	9.12
$L_{\mathrm{gate}}$ (Novelty/entropy calc + EMA)	0.25	0.32	0.40
$L_{\mathrm{CAG}}$ (VRAM Context Cache)	0.80	0.92	1.15
$L_{\mathrm{RET}}$ (HNSW Index + Re-ranking)	26.50	32.10	38.40
$L_{\mathrm{post}}$ (Fusion + Decoder heads)	1.80	1.95	2.20
Total (Cache Path-Low Novelty)	12.50	13.14	14.67
Total (Retrieval Path-High Novelty)	38.20	44.32	49.92

**Table 4:** System components and interfaces. Inputs/outputs, statefulness, and latency criticality.

Module	Input	Output	State	Criticality
Preprocess P	$I_{t}$	${\tilde{I}}_{t}$	stateless	medium
Vision enc. $E_{\theta }$	${\tilde{I}}_{t}$	$v_{t}\in R^{d_{v}}$	parametric	high
Telem. norm. Φ	$S_{t}$	$s_{t}$	stateless	low
Router $G_{\phi }$	$\left(p_{t},v_{t},s_{t}\right)$	$g_{t}$	stateful	high
Cache $M_{\mathrm{CAG}}$	key from $S_{t}$	$c_{t}$	cached	v. high
Retrieval $M_{\mathrm{RET}}$	$\left(v_{t},s_{t}\right)$	$c_{t}$	ext. index	v. high
Fusion Ψ	$\left(v_{t},s_{t},c_{t}\right)$	$z_{t}$	stateless	medium
Decision head $\pi_{\mathrm{dec}}$	$z_{t}$	$a_{t}$	parametric	high

**Table 5:** Encoder configuration. Hyperparameters used to train the hardware-aware Nested U-Net.

Item	Value
Input resolution	512×512 (RGB, fp16 normalized)
Embedding dimension $d_{v}$	512 (L2-normalized)
Backbone Architecture	ResNet-18 (with UNet++ dense skip aggregation)
Base channels	[64,128,256,512]
Normalization	GroupNorm (groups = 32)
Activation	SiLU
Optimizer	AdamW (lr = $1\times 10^{-4}$, wd = $1\times 10^{-2}$)
Loss weights (α,β,γ)	(1.0,0.5,0.3)
Contrastive temperature τ	0.07

**Table 6:** Orchestrator hyperparameters. Calibrated for the Jetson AGX Orin target.

Param	Value	Description/Rationale
λ	0.50	Base novelty threshold
δ	0.10	Hysteresis band to prevent flicker
*m*	5	Min dwell time (frames) ≈42 ms at 120 fps
ρ	0.85	EMA smoothing factor
$T_{\mathrm{cal}}$	1.5	Temperature scaling for calibrated softmax
$\omega_{1..3}$	[0.6,0.2,0.2]	Weights: Entropy, Energy-OOD, Drift
${\hat{L}}_{\mathrm{RET}}^{\left(p99\right)}$	45 ms	Tail-latency guard (conservative cap)
${\hat{L}}_{\mathrm{post}}^{\left(p99\right)}$	3 ms	Conservative post-cost (fusion+heads)

**Table 7:** Hybrid memory configuration. Hyperparameters tuned for Jetson AGX Orin (MaxN mode) to satisfy the 50 ms deadline.

Parameter	Value	Rationale/Constraint
Δσ	10.0 m	Cache bin size ($>v_{\max}B$ margin)
α	0.01	Drift test confidence (99%)
η	0.005	Slow EMA adaptation to filter sensor noise
*w*	60 frames	0.5 s persistence to confirm drift
*k*	5	Soft-voting size (precision versus latency)
$\tau_{s}$	0.07	Softmax temperature for similarity weighting
HNSW *M*	32	Graph connectivity for d=512 vectors
efSearch	64	Caps traversal to bound tail latency
Partitions	Year_Track	Index isolation (domain/track)

**Table 8:** Aspar-Synth-10K anomaly taxonomy and injection parameters. Frequency bands reflect plausible chassis/suspension vibration ranges; values are scenario-calibrated (not an official MotoGP specification).

Class	Phenomenon	Freq. Band	Trigger/Onset	Severity Func.	Samples
y=0	Nominal (track/env)	–	Random	–	4000
y=1	Headshake (front)	6–9 Hz	Accel (>30% thr)	Linear (αt)	1500
y=2	Suspens. chatter	18–24 Hz	Lean ($>45^{\circ }$)	Sigmoid step	1500
y=3	Brake resonance	12–16 Hz	Brake (>15 bar)	Pressure-coup.	1500
y=4	Tire graining	Spatial	Stint (Lap>15)	Exp. accum.	1500

**Table 9:** Edge inference specifications (deployment target).

Parameter	Configuration
Platform	NVIDIA Jetson AGX Orin (64 GB)
Compute	275 TOPS (INT8 sparse, peak)
Memory BW	204.8 GB/s
Runtime	TensorRT 8.x, batch = 1, zero-copy when available
Constraint	50 W TDP, B=50 ms deadline

**Table 10:** Experimental variants (ablation study).

ID	Configuration	Research Question
B0	No-Mem	Is external memory necessary beyond a strong encoder?
B1	Retrieve-Always	What is the latency/energy penalty of continuous retrieval?
B2	Cache-Only	Can circuit redundancy handle novelty without retrieval?
B3	Hybrid Gate	Does uncertainty-based routing balance B1 versus B2?
B4	Hybrid + Hyst.	Does hysteresis reduce routing flicker and stabilize tail latency?
B5	Hybrid + Guard	Does p99-guard improve safety metrics and determinism?
B6	w/o UNet++	Is UNet++ refinement necessary for texture anomalies?

**Table 11:** Main Results (Mean ± Std). End-to-end latency percentiles, deadline miss-rate (L>50 ms), diagnostic performance, and energy consumption. Red indicates budget violations.

Variant	Latency (ms)			Miss	Diagnosis		Efficiency		
	P50	P95	P99	%	F1	AUC	FPS	W	J/F
B0 (No-Mem)	12.4	16.1	18.5	0.0	0.62	0.70	75	28.0	0.37
B1 (Retr)	38.2	95.4	112.1	16.8	0.89	0.94	26	36.0	1.38
B2 (Cache)	13.1	17.5	21.3	0.0	0.71	0.79	72	29.0	0.40
B3 (Hybrid)	16.5	42.1	58.4	2.1	0.84	0.90	55	33.0	0.60
B4 (Hyst)	16.8	39.5	49.2	0.9	0.86	0.91	56	32.5	0.58
**B5 (Ours)**	**16.9**	**38.2**	**46.5**	**0.4**	**0.88**	**0.93**	**58**	**31.5**	**0.54**

**Table 12:** Per-Class F1 Score Analysis. The hybrid architecture yields decisive gains in dynamic/oscillatory classes (chatter, shaking) compared to static baselines. Δ denotes the net improvement of B5 over B0.

Anomaly Class	Dynamics	B0	B5	Δ Gain
Normal (Nominal)	Static	0.93	0.98	+5%
Track Limits	Static	0.92	0.94	+2%
Tire Blistering	Visual	0.78	0.88	+10%
Brake Shaking	12–16 Hz	0.66	0.85	+19%
Susp. Chatter	18–24 Hz	0.61	0.89	+28%

**Table 13:** Operational Burden at High Recall (R≈0.90). B5 maintains high precision where baselines fail. FAR indicates operational noise.

Method	Precision (R≈0.9)	False Alarm Ratio	Operational Status
B0 (No-Mem)	0.44	1.27 (High)	Unusable (Noise > Signal)
B2 (Cache)	0.52	0.92 (Med)	Marginal
B5 (Ours)	0.74	0.35 (Low)	Viable (Signal > Noise)

**Table 14:** Robustness (Macro PR-AUC). While B0 degrades under stress, B5 retains stability comparable to retrieval-only B1 while remaining real-time safe.

Variant	A: Nominal	B: Stress	C: Env. Shift	Stability (Δ)
B0 (No-Mem)	0.72	0.68	0.69	−5.6%
B1 (Retrieval-only)	0.95	0.93	0.92	−3.1%
B5 (Ours)	0.94	0.92	0.91	−3.2%

**Table 15:** B5 scenario profile. Energy correlates with retrieval usage ($\pi_{\mathrm{ret}}$). Nominal laps are most efficient; stress remains feasible under the 50 ms deadline and 50 W cap.

Scenario	$\pi_{\mathrm{ret}}$	P99 (ms)	Miss (%)	FPS	Avg W	J/Frame
A: Qualifying (Nominal)	0.12	24.0	0.0	76	30.5	0.40
B: Mechanical Stress	0.45	46.5	0.4	51	34.5	0.68
C: Environmental Shift	0.23	39.0	0.2	61	31.1	0.51

**Table 16:** Reliability and Thermal Margins. The system respects the real-time deadline (WDT <1%) and thermal envelope across all scenarios. ΔT indicates headroom before thermal throttling triggers (95 °C).

Test Scenario	Retrieval Rate	WDT Drops	Clamp Events	$T_{\mathrm{peak}}$	Margin (ΔT)
A: Qualifying (Nominal)	12%	0.00%	0.10%	78 °C	+17 °C
B: Mech. Stress (High)	45%	0.40%	0.80%	88 °C	+7 °C
C: Env. Shift (Medium)	23%	0.20%	0.35%	82 °C	+13 °C

**Table 17:** Sector-wise Analysis. High-speed sectors allow for cache reuse (low $\pi_{\mathrm{ret}}$), while technical low-speed sectors trigger retrieval to handle uncertainty.

Sector	Avg Speed (km/h)	$\pi_{\mathrm{ret}}$	Mean H
S1 (Main straight)	270	0.05	0.15
S2 (Turn 1 braking)	60	0.68	0.75
S3 (Turn 2 apex)	100	0.45	0.55
S4 (Banked)	160	0.30	0.40
S5 (Back straight)	240	0.08	0.20
S6 (Tight chicane)	55	0.72	0.80
S7 (Fast curve)	180	0.25	0.35
S8 (Finish straight)	280	0.04	0.12

**Table 18:** Retrieval Hygiene. Unfiltered similarity retrieval is polluted by obsolete context. Domain filtering eliminates physics-inconsistent grounding, ensuring retrieved evidence remains mechanically compliant.

Configuration	Rel@1 ↑	Rel@5 ↑	Hallucination Rate ↓	Status
Unfiltered retrieval	0.78	0.72	0.28	Unsafe (Obsolete Physics)
Domain-aware	0.92	0.88	0.00	Compliant

**Table 19:** Reliability Statistics (B5). The system maintains >99.6% availability across regimes. MTBA indicates that drops are rare events.

Scenario	Availability	Drop Rate	Max Burst	MTBA (Frames)
A (Nominal)	100.0%	0.0%	0	∞
B (Mech. Stress)	99.6%	0.4%	2	250
C (Env. Shift)	99.8%	0.2%	2	500

**Table 20:** Graceful Degradation (Safe Mode). Upon thermal trigger, the system sheds retrieval load. Latency drops by half, ensuring the device does not overheat, albeit at reduced classification performance.

State	Architecture	P99 Latency	Thermal Load	Macro-F1
Normal	Hybrid (B5)	46.5 ms	100% (Nominal)	0.88
Safe Mode	Cache-Only (B2)	21.3 ms	60% (Cooling)	0.71

**Table 21:** Claim–evidence matrix. Mapping architectural claims to measured artifacts.

Core Claim	Empirical Artifact (Evidence)	Status
1. Real-time feasibility	Figure 11 (ECDF) and Table 11 show tail latency remains below the 50 ms hard deadline (P99 <50 ms).	Verified
2. Diagnostic gain	Figure 12 (uplift) and Figure 13 (confusion matrix) show gains on dynamic anomaly classes (e.g., chatter).	Verified
3. Fail-silent safety	Figure 21 (burst analysis) and Figure 23 (FSM) confirm deterministic degradation without staleness.	Verified
4. Energy efficiency	Figure 17 (Pareto) and Figure 18 validate the energy reduction relative to always-on retrieval.	Verified

**Table 22:** Key quantitative takeaways (B5). All values are measured on-device on Jetson AGX Orin (50 W cap), consistent with Section 4.

Dimension	Metric	Value
Real-time tail	P99 latency/miss-rate	46.5 ms/0.4%
Diagnostic quality	Macro-F1/Chatter F1	0.88/0.89
Energy viability	Avg power/energy per frame	31.5 W/0.54 J
Fail-silent delivery	WDT abort (Scenario B)/max burst	0.4%/2

**Figure A1:**
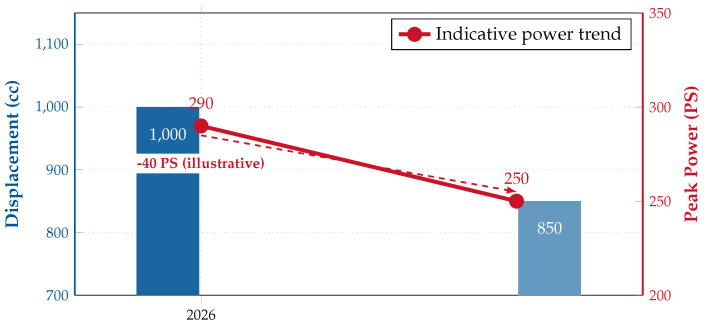
Engine regulation shift (2027). Displacement reduction from 1000 cc to 850 cc is mandated by the 2027 framework. The power values shown are illustrative (not an official specification) and are included only to motivate an expected reduction in acceleration margin. The dashed arrow indicates the projected drop in peak power performance.

**Figure A2:**
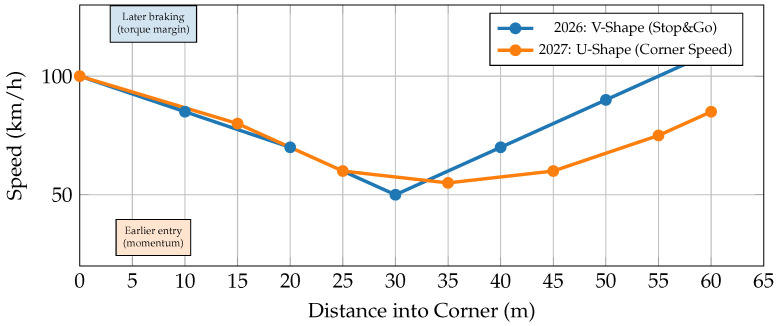
Trajectory evolution (schematic). A conceptual illustration, included only as intuition for how reduced acceleration margin may bias riders toward momentum preservation.

**Figure A3:**
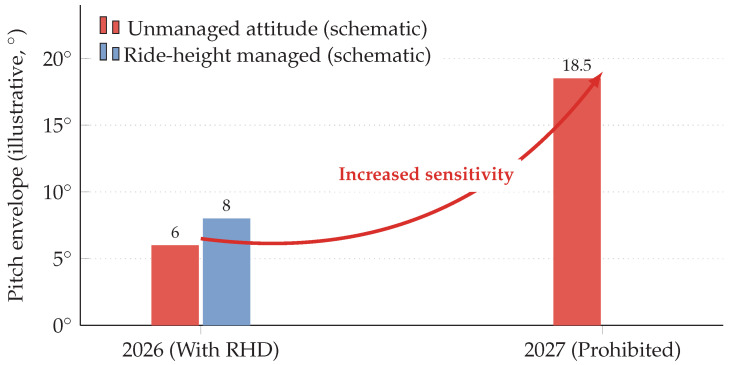
Motivation schematic: attitude sensitivity without RHD. Not an official measurement. Included only to visualize why damping/tire degradations may become critical when mechanical ride-height actuation is prohibited.

## Data Availability

The data presented in this study are openly available in Bucle2D at https://zenodo.org/records/18098196 (accessed on 20 January 2026).

## Appendix A. Regulation-Aware Motivation Figures

This appendix provides additional context on the MotoGP 2027 technical package and includes motivation-only schematics referenced during early drafting. These figures are not used as quantitative evidence in our evaluation; they are included to support intuition about why small degradations in damping, tire state, or stability margins can become operationally critical when mechanical/aero stabilizers are constrained.

### Appendix A.1. Engine Displacement Shift (2027)

The MotoGP 2027 package mandates a reduction in engine displacement to 850 cc (with a 75 mm maximum bore) while simultaneously restricting aerodynamics and prohibiting mechanical ride-height and holeshot devices [1,2,3]. The key point for this paper is not the propulsion subsystem itself but the downstream shift in on-track envelopes that changes the distribution of nominal versus high-uncertainty visual states.

### Appendix A.2. Trajectory Evolution Schematic

With altered aerodynamic support and reduced acceleration headroom, riders may be biased toward momentum preservation and smoother cornering lines. This matters computationally because it changes the stability of visual reference points (braking markers, apex approach textures, exit trajectories) that can be exploited as static context by caching mechanisms.

### Appendix A.3. Motivation Schematic: Attitude Sensitivity Without Ride-Height Devices

The prohibition of mechanical ride-height devices removes one of the practical actuation mechanisms teams used to shape attitude transitions during launch and acceleration. In racing motorcycles, attitude transitions and vibration modes are tightly coupled; classical studies describe how racing chatter emerges from interactions between tire dynamics, suspension compliance, and chassis modes [4,5]. The figure below is therefore presented strictly as a motivation schematic: it does not claim official pitch numbers, but it visualizes why small degradations in damping or tire state can become operationally critical when mechanical ride-height actuation is prohibited.
